# Targeting macrophage priming by polyphyllin VII triggers anti-tumor immunity via STING-governed cytotoxic T-cell infiltration in lung cancer

**DOI:** 10.1038/s41598-020-77800-w

**Published:** 2020-12-07

**Authors:** Jinglu Yu, Haibin Deng, Zhenye Xu

**Affiliations:** 1 Department of Oncology, Shanghai University of Traditional Chinese Medicine Longhua Hospital, Shanghai, 200032 China; 2 Shanghai University of Traditional Chinese Medicine, Shanghai, 200030 China

**Keywords:** Cancer, Immunology

## Abstract

Stimulator of interferon genes (STING) controlled innate immune pathway is essential for host defense against pathogenic infection and effective anti-tumor adaptive immunity initiation. Although macrophages transformed across diverse phenotypes play crucial roles in anti-tumor immune response, events determining this transformation and the host-intrinsic role of STING in this process remain controversial. Here we report how STING signaling acts as a key switch to dominate the gene expression patterns of macrophage transformation for promoting priming and releasing immunosuppression. Furthermore, polyphyllin VII, a potential STING agonist, exerts anti-tumor efficacy upon macrophages priming and subsequent cytotoxic T lymphocytes intratumoral infiltration. Meanwhile, the simultaneous PD-L1 amplification on macrophages in response to PP VII is also ruled by STING, thus PP VII may benefit immune-checkpoint blockade therapy for combining. Moreover, PP VII suppresses carcinogenesis upon restraining the immunosuppressed macrophage transformation. This is due to the boosted STING that negatively regulates a STAT3 propagated crosstalk between immune cells and tumor cells. Overall, PP VII-stimulated STING in macrophages provides a paradigm for anti-tumor, and if possible, anti-infection immunotherapy.

## Introduction

Despite considerable advances in treatment of lung cancer in recent years, the prognosis of patients remains very poor^1,2^. The programmed death 1 receptor/ programmed death-ligand 1 (PD1/PDL1) blockade therapy have shown unprecedented durable response in some lung cancer patients, which seems to have brought dawn. However, only a minority of patients benefit from such therapies^3–5^. It is increasingly recognized that the tumor immune microenvironment (TIME) determines the outcome of immunotherapy^6–9^. Macrophages are a major component of the leukocyte infiltrated in the TIME^10^. They are transformed upon exposed to multiple signals of local or systemic origin thus endow them environmentally sensitive cells with high plasticity, then subsequently give response to the extracellular milieu in shaping the immune environment^11^. Given that they are extremely versatile cells performing pleiotropic cellular programs, such as phagocytosis of pathogens, processing and presentation of antigens, secretion of chemicals to recruit and reshape other cells, therefore considered a promising Intervention target for transforming the immunosuppressive TIME into an immune activated one^10–13^.

The paradigms employed most intensely in functional and molecular studies on macrophages in vitro have focused on exposing them to potent polarizing ligands, like Interferon-gamma (IFN-γ) for stimulating classic activated (M1-type) macrophages while interleukin 4 (IL-4) for alternative activated (M2-type) macrophages^14,15^. M1 macrophages marked by IFN stimulated genes (ISGs) display an immune-activated phenotype involved in enhanced inflammatory response and antigen presentation. Conversely, M2 macrophages refer to anti-inflammatory functions linked to wound healing and tissue repair^16,17^. M1 macrophages promote tumor rejection by secreting proinflammatory cytokines to prime the tumor immune microenvironment, while M2 macrophages are generally considered to closer to the tumor-promoting tumor-associated macrophages (TAMs) relevant to immunosuppression^10,12,13,16^. Extensive previous studies had discovered meaningful but fragmented pathway in regulating macrophages transformation, so it is still wondered whether there exists a key switch for dominating the direction of macrophage transformation.

The Cyclic GMP-AMP synthase (cGAS)—stimulator of interferon genes (STING) pathway is central for host resisting pathogenic infection^17,18^. Emerging evidence indicated that it is also the major innate immune pathway related to generation of spontaneous antitumor T cell response^19–22^. STING is highly expressed in macrophages, the link between innate and adaptive immunity, where its host-intrinsic role in linking primary and acquired anti-tumor response remains controversial.

Previous researches noticed that the lack of cGAS or STING abrogated anti-tumor effect of programmed death ligand-1 (PD-L1) treatment^23,24^. It is worth noting that PD-L1 on macrophages is expressed more frequently than that on tumor cells in patients with lung cancer and some other carcinomas. Given that macrophages may be the major cellular source for maintaining PD-L1 expression, we have to focus on the unknown mechanism responsible for PD-L1 regulation in macrophages^6–9^. Interestingly, IFN- γ is more than the classic stimulator of M1 macrophages, but generally considered the most prominent soluble inducer of PD-L1^6^. It means that when macrophages are transformed into M1 type, it is likely to be accompanied by PD-L1 expression^25^. Therefore, the question is whether there is an intersection between the mechanism of switching macrophages transformation and inducing PD-L1 production.

The Stat3 pathway plays a key point in mediating the crosstalk between tumor cells and immune cells to provoke tumorigenesis and immunosuppression, since its constitutively activation mutually propagates among different cell subsets in the TME^26,27^. Recent studies showed that STING negatively regulates STAT3 feedforward between host cells and tumor cells^28^, vice versa, inhibition of Stat3 enhances the activity of STING in macrophages^29^. Here we focus on whether STING is involved in Stat3 activation in macrophages that contributes to malignant transformation.

Polyphyllin refer to a series of steroidal saponins isolated from Rhizoma Paridis, the key player in traditional Chinese medicine for anti-tumor. It exhibits extensive anti-tumor effects via apoptosis induction and angiogenesis inhibition in various cancers^30–33^, including Lung cancer^34^. However, the effect of polyphyllin on TIME, especially in macrophage transformation, remains unclear. In the present study, we screened and determined one of them, Polyphyllin VII (PP VII), which manages macrophages transformation to suppress carcinogenesis in vitro, and may subsequently edit TIME in vivo.

Here we report that STING pathway is essential for macrophages priming by triggering type I IFN production thus boosting downstream ISGs expression. We found polyphyllin VII exerts antitumor efficacy upon macrophages priming in vitro and subsequent cytotoxic T lymphocytes intratumoral infiltration in vivo, which is governed by STING signaling. Meanwhile, the simultaneous PD-L1 amplification on macrophages in response to PP VII also depends on STING. Furthermore, PP VII suppresses malignance of tumor cells by restraining the immunosuppressive macrophage transformation, which is also managed by STING, via the prevention of activated-STAT3 propagating between macrophages and tumor cells.

## Results

### PP VII mediates macrophage transformation associated with STING

**Figure 1:**
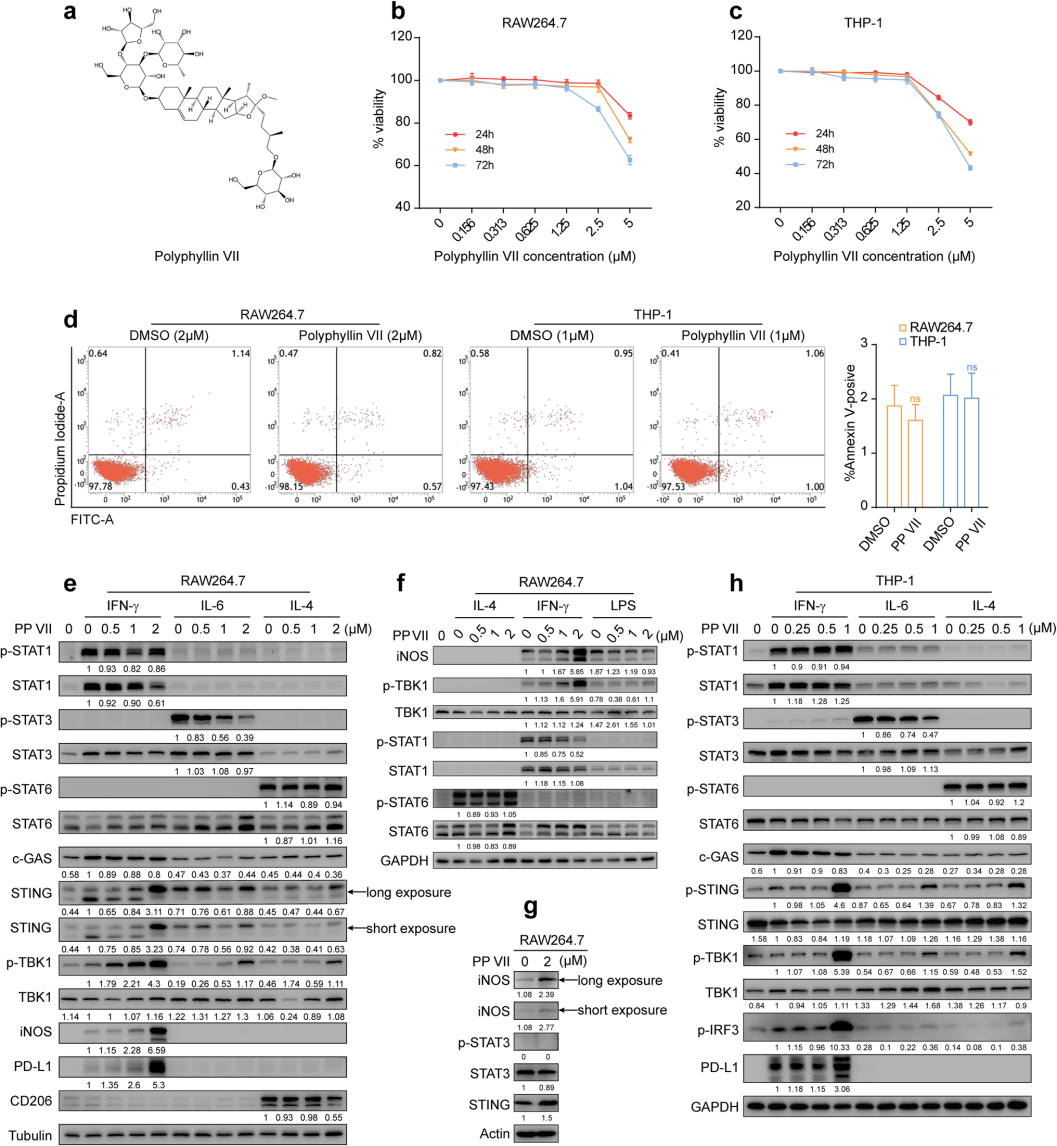
PP VII mediates macrophage transformation associated with STING. (**a**–**h**) PP VII, a small molecular compound (**a**). CCK8 assays show the cell viability of RAW 264.7s (**b**) and THP-1s (**c**) incubated with PP VII at indicated concentration for 24, 48, 72 h separately. Data are depicted as mean + SD of triple samples from one representative experiment of two. The effect of PP VII on cell death for 24 h qualified by apoptosis in RAW 264.7s and THP-1s (**d**). Data are mean + SD of triplicate samples. *ns* no significant. Immunoblots analysis of RAW 264.7s and THP-1s stimulated by indicated cytokines for 24 h following a 3 h of PP VII pretreatment with indicated antibodies (**e**,**f**,**h**). RAW 264.7s left unstimulated only treated by PP VII for 24 h (**g**). Representative immunoblots form 2–3 independent experiments are depicted. If indicated, cells were stimulated with 100 ng/ml IFN-γ or 50 ng/ml IL-6 for 24 h. THP-1 cells were differentiated with PMA before any treatment and, unless stated otherwise.

We observed the toxicity of PP VII (Fig. 1a) to macrophages on cck-8 assay to screen appropriate concentrations for treatment. We found that 0 ~ 2.5 µm and 0 ~ 1.25 µm PP VII separately had no toxic effects on RAW264.7s and THP-1s within 72 h incubation in general, except a slight decrease in cell viability of RAW264.7s after incubation with PP VII for 72 h (higher than 85%) (Fig. 1b,c). These results were further confirmed by the fact that 2 µm and 1 µm PP VII did not induce cell death of RAW264.7s and THP-1s respectively for incubation 24 h (Fig. 1d). Conclusions above were drawn after comparing with the equivalent volume of DMSO.

We next investigated the transformation in macrophages and relevant inflammatory signals in response to different stimuli. The data showed that 2 µm PP VII remarkably potentiated the expression of iNOS, the major M1 marker induced by IFN- γ in RAW264.7 cells (not expressed in THP-1s), in protein level (Fig. 1e). The treatment also augmented IFN-γ induced PD-L1 production in RAW264.7s and THP-1s (Fig. 1e,h). Furthermore, this treatment led to potent activation of STING signaling^35,36^, the starting point of signal transduction is STING instead of cGAS (Fig. 1e,h). Nevertheless, this effect showed no relationship with the JAK-STAT1 cascade downstream of IFN-γ receptor (Fig. 1e,h). Besides, PP VII negatively regulated CD206, a main M2 marker induced by IL-4 in RAW264.7s. The down-regulation was accompanied by STING activation, albeit to a lesser degree than the M1 model, and did not refer to the transcription of STAT6 downstream of IL-4 (Fig. 1e,h). Lipopolysaccharide (LPS), another commonly used stimulator for M1 macrophage polarization transmitted by Toll-like receptor 4 (TLR4)^37^, did not induce PD-L1 expression, which is distinct from IFN-γ. Surprisingly, PP VII combined with LPS did not augment M1 macrophage polarization as it did with IFN-γ (Fig. 1f). IL-6-STAT3 is commonly referred to immunosuppression and tumorigenesis^38,39^, addition PP VII shapely diminished STAT3 transcription stimulated by IL-6, along with upregulated protein level of STING (Fig. 1e,h). Compared to combination with cytokines, PP VII alone slightly induced iNOS and STING expression, however, had no influence on STAT3 activity (Fig. 1g). Collectively, these data suggest that PP VII activates the STING/TBK1/IRF3 pathway and subsequently augments M1 macrophage polarization, as well as suppresses Stat3 phosphorylation, this correlates with STING protein expression rather than induction of DNA damage.

### PP VII modulates macrophage transformation in a time independent manner according to STING activation

**Figure 2:**
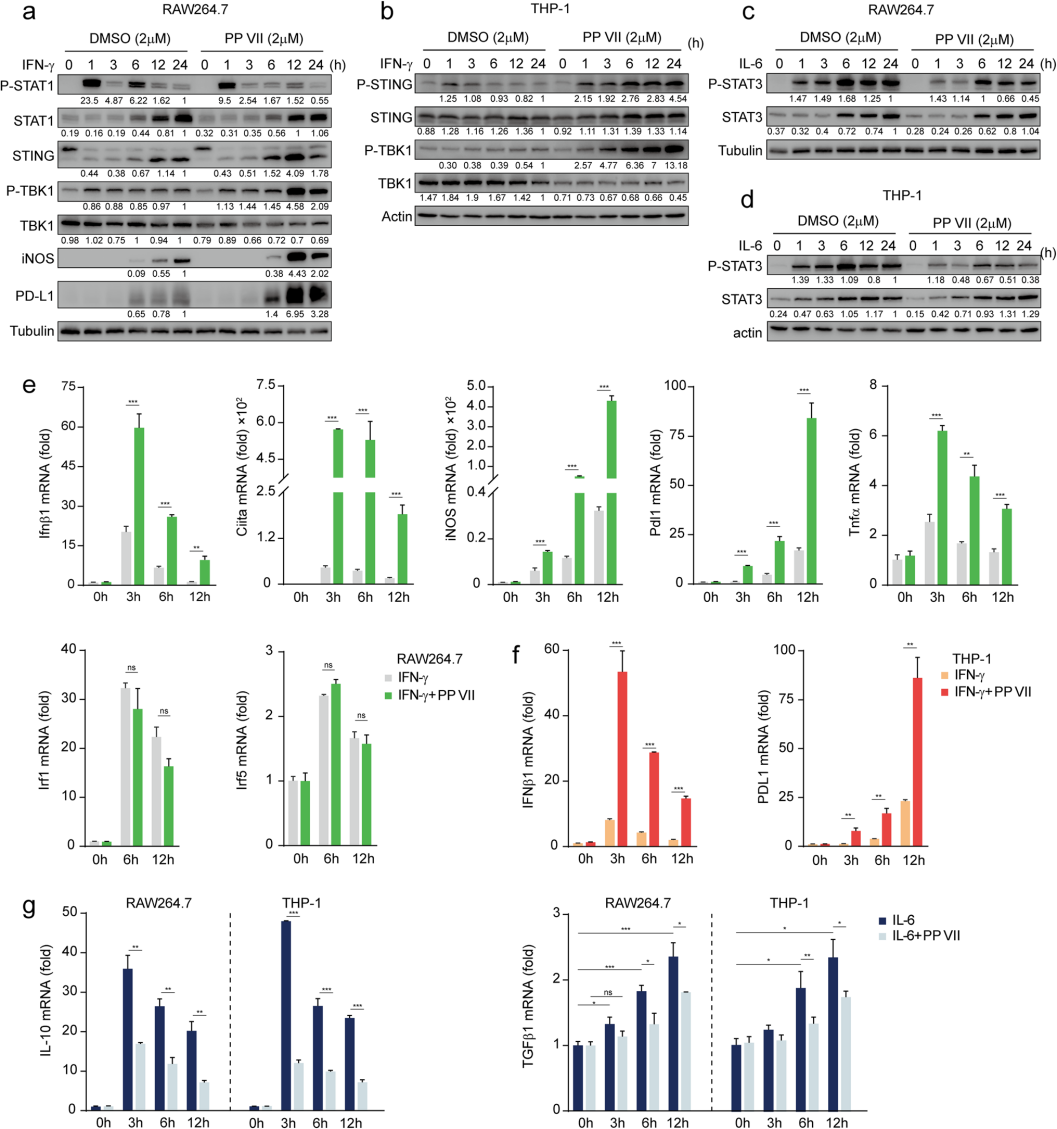
PP VII modulates macrophage transformation in a time independent manner according to STING activation. (**a**–**g**) RAW 264.7s and THP-1s were pretreated with or without PP VII and stimulated with IFN-γ or Il-6 for indicated time. One immunoblot of two is shown (**a**–**d**). Fold change of samples was qualified by qRCR and is depicted as mean + SD of triplicates from one representative experiments of two (**e**–**g**). ***p < 0.001, **p < 0.01, *p < 0.05, *ns* no significant.

To confirm whether the effect of PP VII targeting on macrophage transformation leveraged STING as a key switch, we tested the changes of each component at different time points after intervention. We observed that PP VII promoted the protein level of iNOS and PD-L1 induced by IFN-γ in RAW264.7s, consistent with STING pathway activation (Fig. 2a). The activity of p-STING and p-TBK1 was also efficiently enhanced in THP-1s in a time-dependent manner when PP VII was added prior IFN-γ, compared to IFN-γ alone (Fig. 2b). PP VII reduced STAT3 phosphorylation induced by IL-6 both in RAW264.7s and THP-1s (Fig. 2c,d). Despite the action of PP VII is not strictly time-dependent, it indeed works at all time points.

To further clarify the regulatory mechanism, we detected the changes of mRNA level after treatment. PP VII amplified the expression of Ifnβ gene downstream of STING induced by IFN-γ in a time-dependent manner (Fig. 2e). Ciita, the gene encoding MHC (major histocompatibility complex) II molecule, represents the antigen presentation capability of macrophages and is also a key M1 marker^15,16^. The mRNA levels of iNOS, Pdl1, Ciita, Tnf-α induced by IFN-γ in RAW264.7s were enhanced in a time-dependent manner in the presence of PP VII (Fig. 2e). We observed a similar phenomenon in THP-1s, PP VII promoted the production of Ifnβ and Pdl1 genes time-dependently, compared with IFN-γ alone (Fig. 2f). Together, these data demonstrated that PP VII promoted M1 macrophage transformation, as well as activated STING signaling. The Ifnβ gene peaked at 3 h after treatment, however, iNOS and PD-L1 were delayed and peaked at 12 h (Fig. 2e), suggesting that iNOS and Pdl1 may be downstream of Ifnβ. Interestingly, Irf1 and Irf5, the traditional M1 genes transcribed by p-STAT1 had no changes in our system (Fig. 2e). These results further indicated that PP VII potentiated M1 macrophage transformation associated with STING instead of STAT1. The highlight immunosuppressive cytokines IL-10 and Tgfβ, relating to proliferation and epithelial mesenchymal transformation (EMT)^40–42^, can be secreted by tumor-associated macrophages^10,12,13^and modulated by STAT3^43^. PP VII blocked IL-10 expression induced by IL-6 while reduced Tgfβ production upregulated by IL-6 in RAW264.7s and THP-1s time-dependently (Fig. 2g). Together, these data indicate that PP VII primes macrophages to transform to immune-activated M1 type and restricts their immunosuppressive transformation, which may be achieved by leveraging STING.

### PP VII regulates macrophage transformation determined by STING

**Figure 3:**
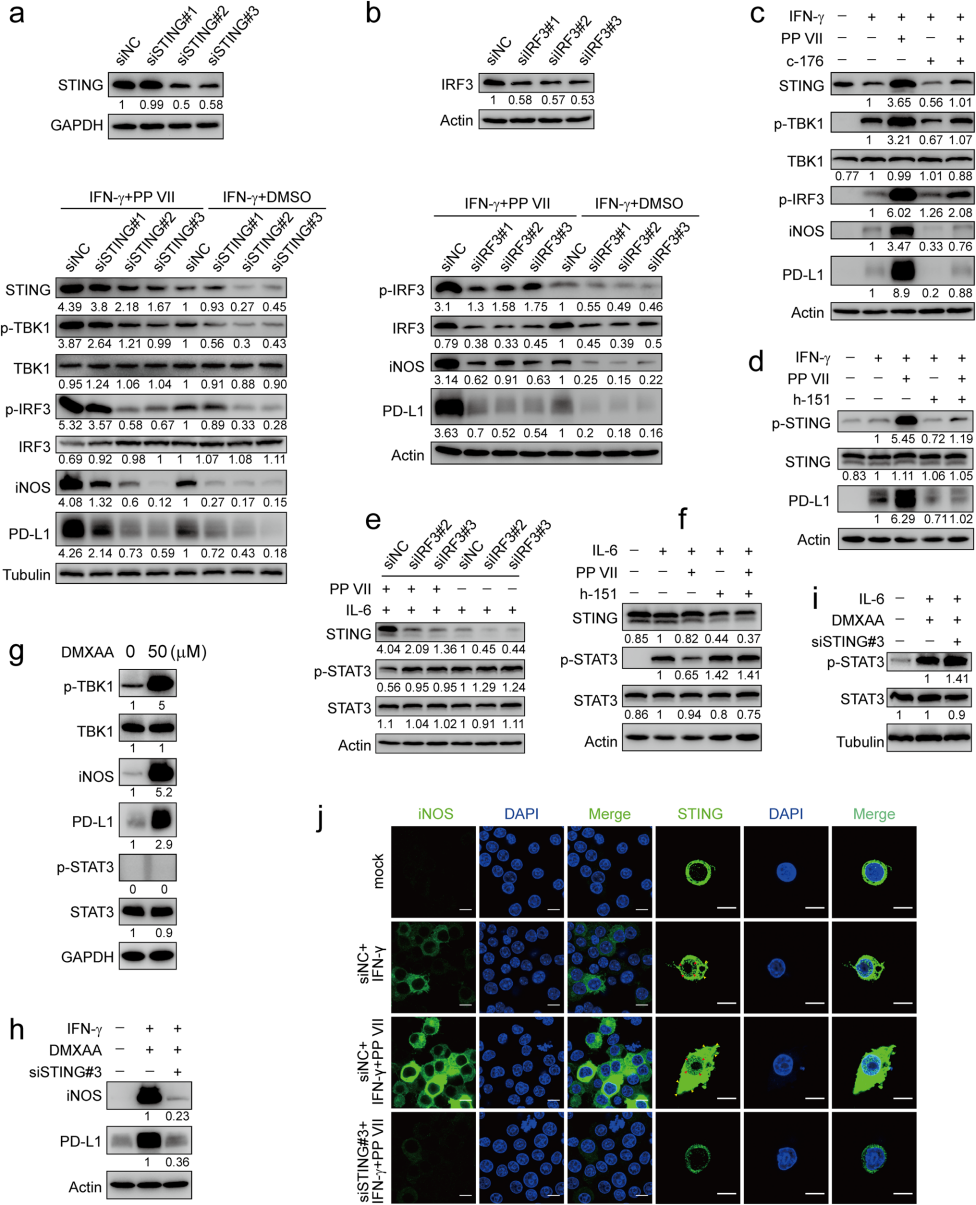
PP VII regulates macrophage transformation determined by STING. (**a**–**j**) RAW 264.7s were transfected with siSTING and siIRF3. 48 h later, cells were stimulated with IFN-γ or IL-6 in absence or presence of PP VII and analyzed by immunoblot (**a**,**b**,**e**). RAW 264.7s and THP-1s with or without PP VII were pretreated with indicated inhibitors and stimulated as in a (**c**,**d**,**f**). RAW 264.7s were treated with DMXAA for 3 h (**g**). siSTING-transfected RAW 264.7s were treated with DMXAA prior to indicated stimulation (**h**,**i**). One representative blot of two independent experiments is shown. siSTING transfected RAW264.7s were pretreated with PP VII for 3 h or left untreated and primed with IFN-γ. Representative confocal images from one experiment of two are depicted (**j**). Scale bars depict 10 µm.

We next generated STING-knockdown RAW264.7 cells using STING-specific siRNAs and observed that the protein level of iNOS and PD-L1 induced by IFN-γ in the presence of PP VII or not was sharply decreased in STING-knockdown RAW264.7s, the same as the activity of TBK-1 kinase and IRF3 transcription downstream of STING. Moreover, Addition PP VII indeed enhanced the STING cascade, compared with IFN-γ alone (Fig. 3a). These conclusions were further confirmed by the facts that once siIRF3 transfected in RAW264.7s to silence STING signaling, iNOS and PD-L1 will be significantly down-regulated (Fig. 3b). Similar results were obtained When STING in RAW264.7s and THP-1s was separately blocked by specific inhibitor C-176 and H-151^44^ (Fig. 3c,d). Next, we found that IL-6 induced STAT3 activity was enhanced in STING-knockdown RAW264.7s and STING-blocked THP-1s. Consistent with previous findings, PP VII inhibited the IL-6 induced transcription of STAT3 by activating STING, the inhibition was reversed in STING-silenced macrophages (Fig. 3e,f). Then we assessed the impact of murine STING-specific agonist DMXAA on RAW264.7s transformation. DMXAA alone or combined with IFN-γ increased the protein level of iNOS and PD-L1, but had no effect on STAT3 phosphorylation without IL-6 (Fig. 3g,h). The promotion of DMXAA on M1 macrophage polarization could be reversed by STING knocked down (Fig. 3h). STAT3 phosphorylation induced by IL-6 was upregulated in siSTING-transfected RAW264.7s in the presence of DMXAA (Fig. 3i).

Photos taken by confocal microscope showed that iNOS appeared in RAW264.7s under IFN-γ stimulation, accompanied by STING activation displaying as the gathering of perinuclear puncta indicated by red arrowheads. The fluorescence intensity of iNOS was drastically enhanced while more pronounced perinuclear puncta were detected (red arrowheads) in the presence of PP VII. However, once STING was silenced, the iNOS enhancement by PP VII was subsequently reversed (Fig. 3j). Interestingly, macrophages changed shape with the extent of STING activation, as shown by gradually extending their tentacles outward from the perfect circle (indicated by yellow arrowheads) until PP VII was added, eventually forming a typical long-spindle M1 shape. These data collectively confirmed that STING cascade is essential for M1 macrophage transformation and macrophage-PD-L1 induction primed by PP VII, as well as STAT3 inactivated by PP VII.

### PP VII primes macrophage to an immune activated pattern dependent on STING

**Figure 4:**
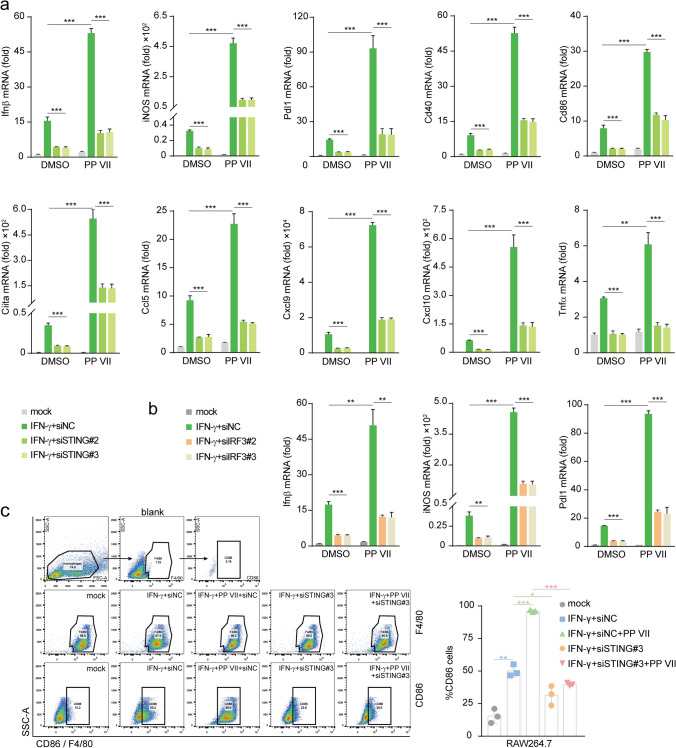
PP VII enhances macrophage priming to an immune activated pattern dependent on STING. (**a**–**c**) siSTING (**a**) and siIRF3 (**b**) transfected RAW 264.7s were pretreated with or without PP VII and primed with IFN-γ for RT-qPCR analysis. CD86 expression was qualified by FACs analysis from Indicated cells that were stimulated as in a (**c**). Data are depicted as mean + SD of triplicate samples from two independent experiments. ***p < 0.001, **p < 0.01, *p < 0.05, *ns* no significant.

To further demonstrate how PP VII regulates the effect of STING cascade on macrophage priming thus enhances M1 macrophage transformation, next we detected the changes in related genes during this process. Ifnβ, the major effector gene of STING signaling, has been reported to induce the production of chemokines Ccl5, Cxcl9, and Cxcl10^19–21^. Since these chemokines are key mediators for the chemotaxis of CD8^+^ T lymphocytes, we investigated their expression during M1 macrophage transformation targeted by PP VII. PP VII treatment caused significantly upregulation of IFNβ, Ccl5, Cxcl9, and Cxcl10 genes, as well as a battery of ISGs, including iNOS, PD-L1, CD40, CD86, CIITA, TNFα, induced by IFN-γ in RAW264.7s. Meanwhile, both their expression under IFN-γ stimulation and enhancement upon addition PP VII were reversed by STING knockdown (Fig. 4a). The mRNA of Ifnβ, iNOS and Pdl1 induced by IFN-γ and their augment by adding PP VII were abrogated in siIRF3 transfected RAW264.7s as well (Fig. 4b). CD86, a costimulatory molecule expressed on macrophages, to optimize antigen production, is considered another main marker of M1 macrophage^16^. Data of fluorescence-activated cell sorting (FACS) showed that CD86 was low expressed in resting RAW264.7s and could be stimulated by IFN-γ, then sharply amplified by addition PP VII. However, CD86 failed to be stimulated and augmented in STING-knockdown RAW264.7s under the above treatment (Fig. 4c).

**Figure 5:**
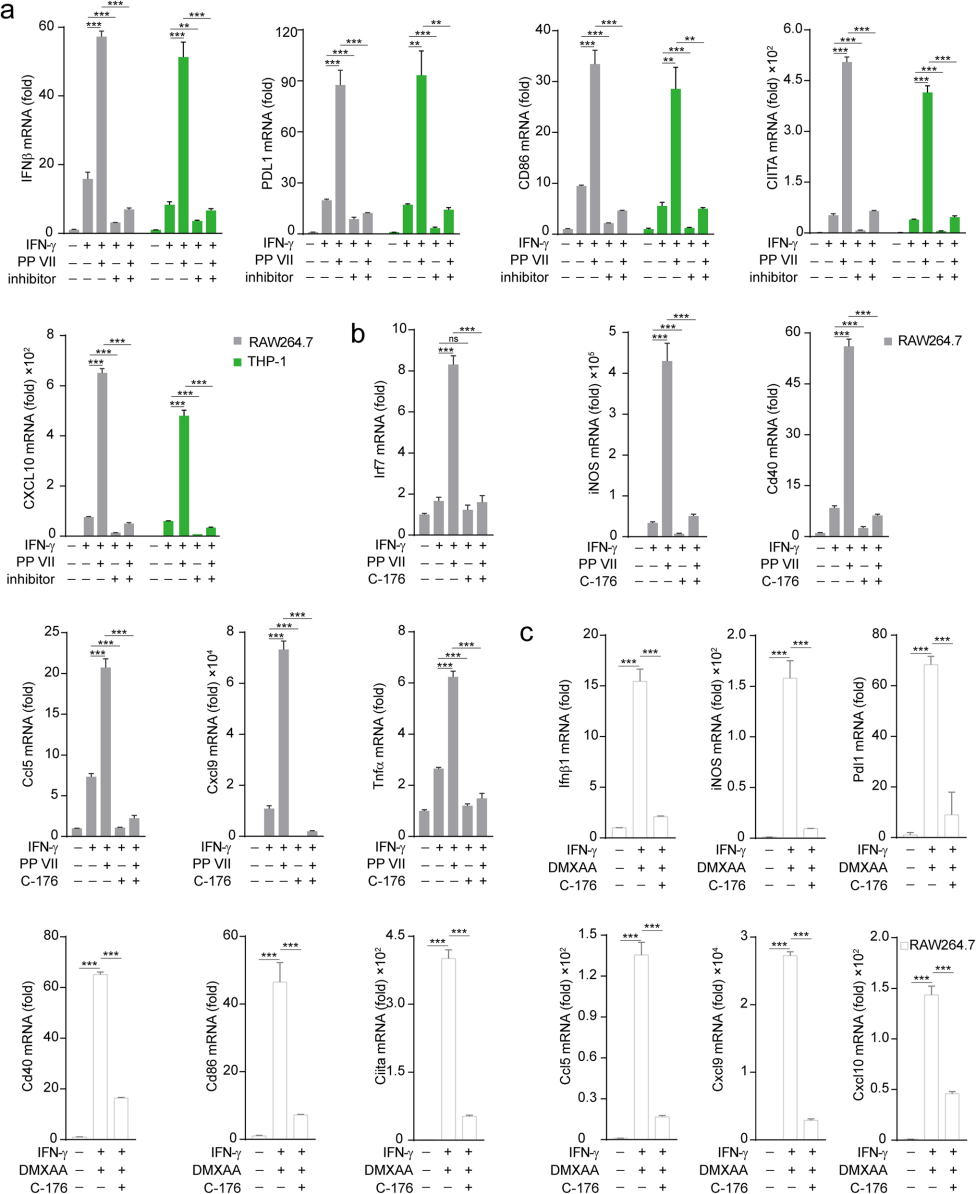
PP VII boosts macrophage activity relying on STING. (**a**–**c**) RAW 264.7s and THP-1s were pretreated with C-176 and H-151 respectively then primed with IFN-γ in absence or presence of PP VII (**a**,**b**). RAW 264.7s with DMXAA pretreatment were primed with IFN-γ following inhibitor incubation (**c**). RT-qPCR analysis of triplicates from two independent experiments is shown as mean + SD. ***p < 0.001, **p < 0.01, *p < 0.05, *ns* no significant.

Similarly, the elevation of IFN-γ responsive genes by adding PP VII was abolished when STING is blocked by C-176 in RAW264.7s or by H-151 in THP-1s (Fig. 5a,b). We also founded that the expression of ISGs and chemokines was efficiently elevated by DMXAA treatment then reversed by STING blocking in RAW264.7s (Fig. 5c). Taken together, our data indicate that PP VII drastically enhances the transcription of chemokines and ISGs in macrophages via targeting STING, thereby triggering their transformation into immune activated pattern.

### PP VII-primed macrophages affect the malignance of tumor cells

**Figure 6:**
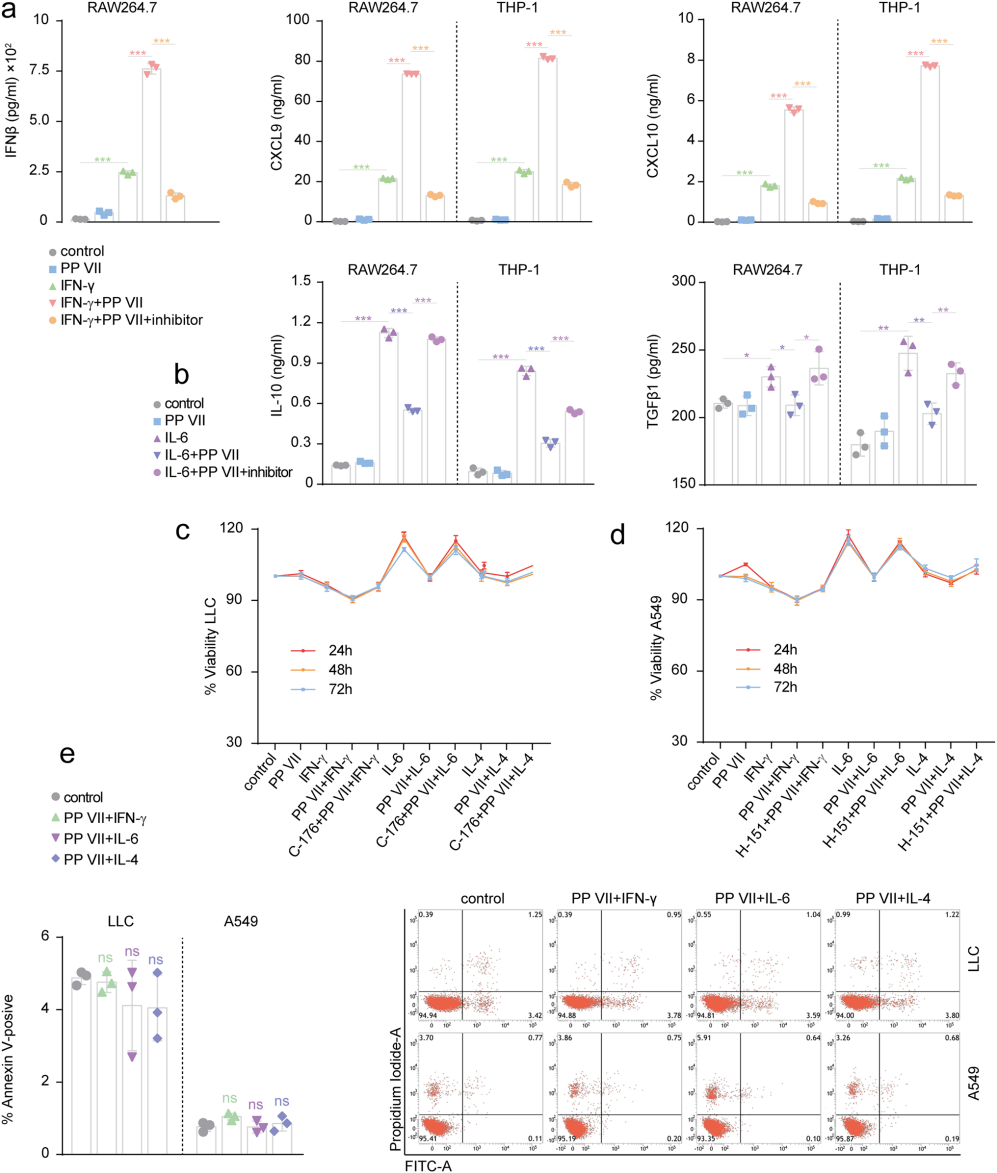
The effect of PP VII-primed Macrophages on the growth and apoptosis of tumor cells. (**a**–**e**) RAW264.7s and THP-1s pretreated with indicated inhibitors were stimulated with IFN-γ (**a**) and IL-6 (**b**) in absence or presence of PP VII. Cytokines secretion was qualified by ELISA and depicted as mean + SD of triplicate samples from two experiments. ***p < 0.001, **p < 0.01, *p < 0.05. LLCs (**c**) and A549s (**d**) were incubated with indicated macrophage CMs for 24, 48, 72 h respectively. Cell viability was qualified by CCK8 and is shown as mean + SD of triplicate samples from one representative experiments of two. The effect of indicated macrophage CM on cell death for 24 h was qualified by apoptosis in LLCs and A549s (**e**). Data are shown as mean + SD of triplicate samples.

Next, we observed the changes of cytokines secreted by transformed macrophages regulated by PP VII and their effects on tumor cells. Addition PP VII resulted in more production of IFNβ, CXCL9 and CXCL10 protein in response to IFN-γ in RAW264.7s and THP-1s. C-176 and H-151 efficiently blocked the elevation of PP VII on IFN-γ responsive proteins (Fig. 6a). And the suppression of PP VII on IL-10 and TGFβ protein secreted by RAW264.7s and THP-1s was reversed by STING blocking (Fig. 6b).

To investigate the impact of PP VII on functional macrophage-tumor cell crosstalk, we correspondingly treated LLCs and A549s with cognate CM. Incubation with CM derived from PP VII-treated M1 macrophages suppressed the survival of LLCs and A549s, compared with these tumor cells treated with naive macrophage CM even M1 macrophage CM. Meanwhile, IL-6-transformed macrophage CM promotes the viability of tumor cells, and addition PP VII inhibited this effect. It suggests that pretreating macrophages with PP VII before their transformation promotes the proliferation-inhibiting effect of IFN-γ transformed macrophage CM on tumor cells, while inhibited the proliferation-promoting effect of IL-6-transformed macrophages CM. But all that were abrogated when CMs were collected from STING-blocked macrophages. Besides, IL-4-treated CM derived from STING-blocked macrophages seemed slightly increase the viability of LLCs and A549s (Fig. 6c,d). It resulted in no apoptosis in LLCs and A549s by incubation with CMs derived from transformed macrophages treated by PP VII (Fig. 6e).

**Figure 7:**
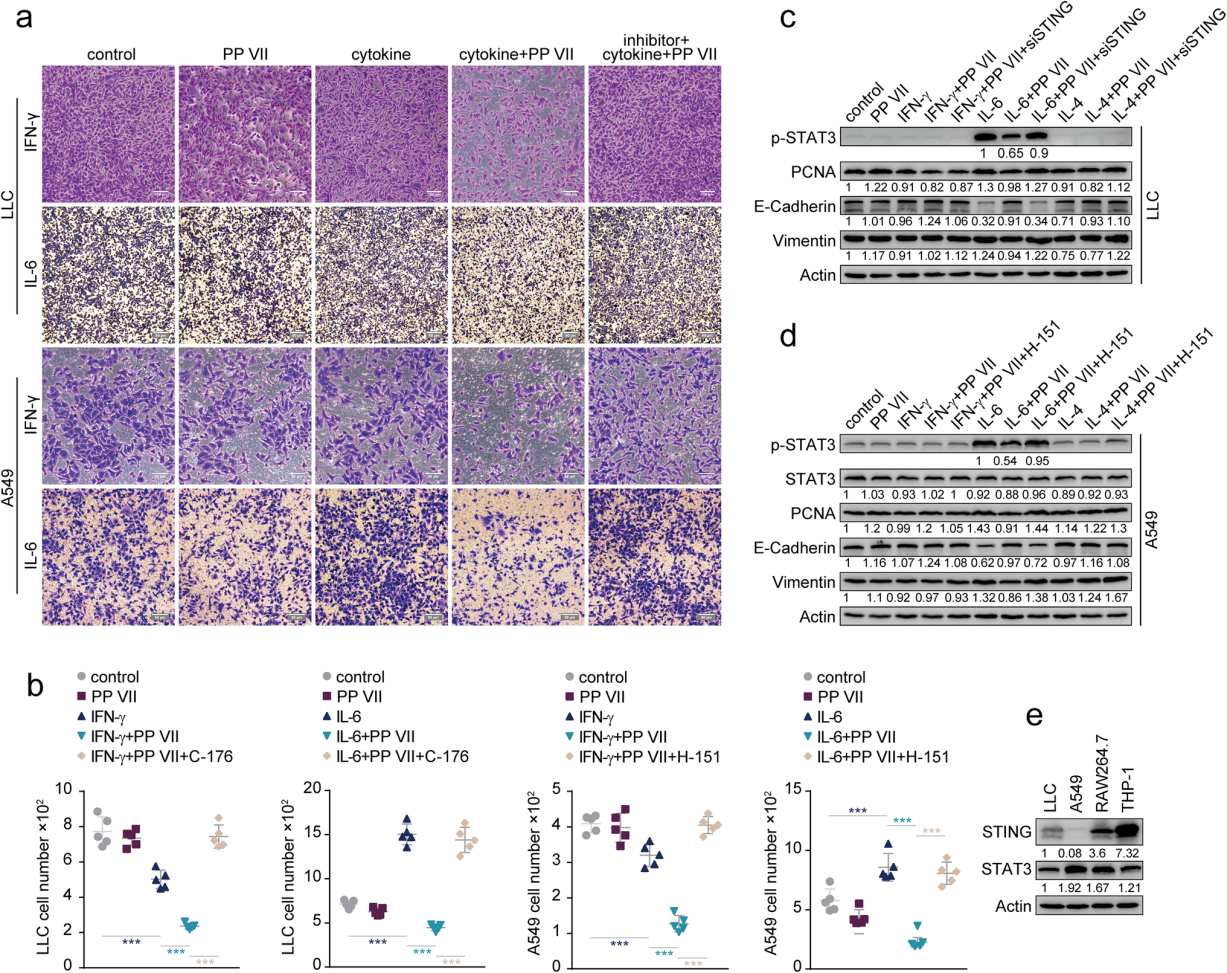
PP VII-primed Macrophages affect the invasion of tumor cells by regulating STAT3 crosstalk. (**a**–**e**) LLCs and A549s were incubated with indicted macrophage CMs within 24 h. Invasion was qualified by transwell assays and is depicted as mean + SD of five random fields from triplicate samples (**b**). Representative micrographs from one experiment of two are shown (**a**). Scale bars denote 50 or 100 µm. ***p < 0.001. Immunoblotting of LLCs and A549s treated for 24 h as above (**c**,**d**). Indicated cells were analyzed by Immunoblot (**e**). Representative blot from one of two experiments is shown.

We further examined whether the CMs affected invasion of tumor cells and found that PP VII-treatment further reduced the number of invasive LLCs and A549s Incubated with M1 macrophage-CM while decreased the growing number of invasive tumor cells promoted by IL-6-transformed CM within 24 h, but not for CMs from STING-blocked macrophages (Fig. 7a,b). However, we observed no significant impact of IL-4-CM on the number of invaded cells (data not shown).

Meanwhile, we detected that Stat3 was drastically activated only in tumor cells treated by IL-6-CM, and addition PP VII prior IL-6 in macrophages during preparing CM prevented the provocation, but not for STING-knockdown macrophages (Fig. 7c,d). Since IL-6/STAT3/IL-10/TGF-β plays pivotal role in carcinogenesis and EMT^39–43,45–48^, to verify whether this axis is involved in malignant transformation of tumor cells regulated by transformed macrophage-CMs, we detected the changes of several major indicators in this process. The expression of Vimentin (an indicator of EMT) and PCNA (related to active proliferation) was in line with the trend of STAT3 phosphorylation regulated by STING in response to PP VII. But E-cadherin, a negative regulator of EMT, had an opposite pattern under the same condition (Fig. 7c,d). Given to Stat3 is negative regulated by STING in macrophages, we wondered whether this relationship also exists in tumor cells. However, our data showed that STING was quite low expressed in LLCs, and almost hardly expressed in A549s, unlike heavily expressed in macrophages (Fig. 7e). These data together indicate that PP VII promotes proinflammatory cytokines secreted by M1 macrophage and inhibited STAT3 activation in tumor cells driven by IL-10 and TGF-β secreted by STAT3-activated macrophages upon targeting STING, thereby suppressing proliferation and invasion of tumor cells in a paracrine manner.

### PP VII induces CD8^+^ T cell infiltration and anti-tumor efficacy dependent on STING activation

**Figure 8:**
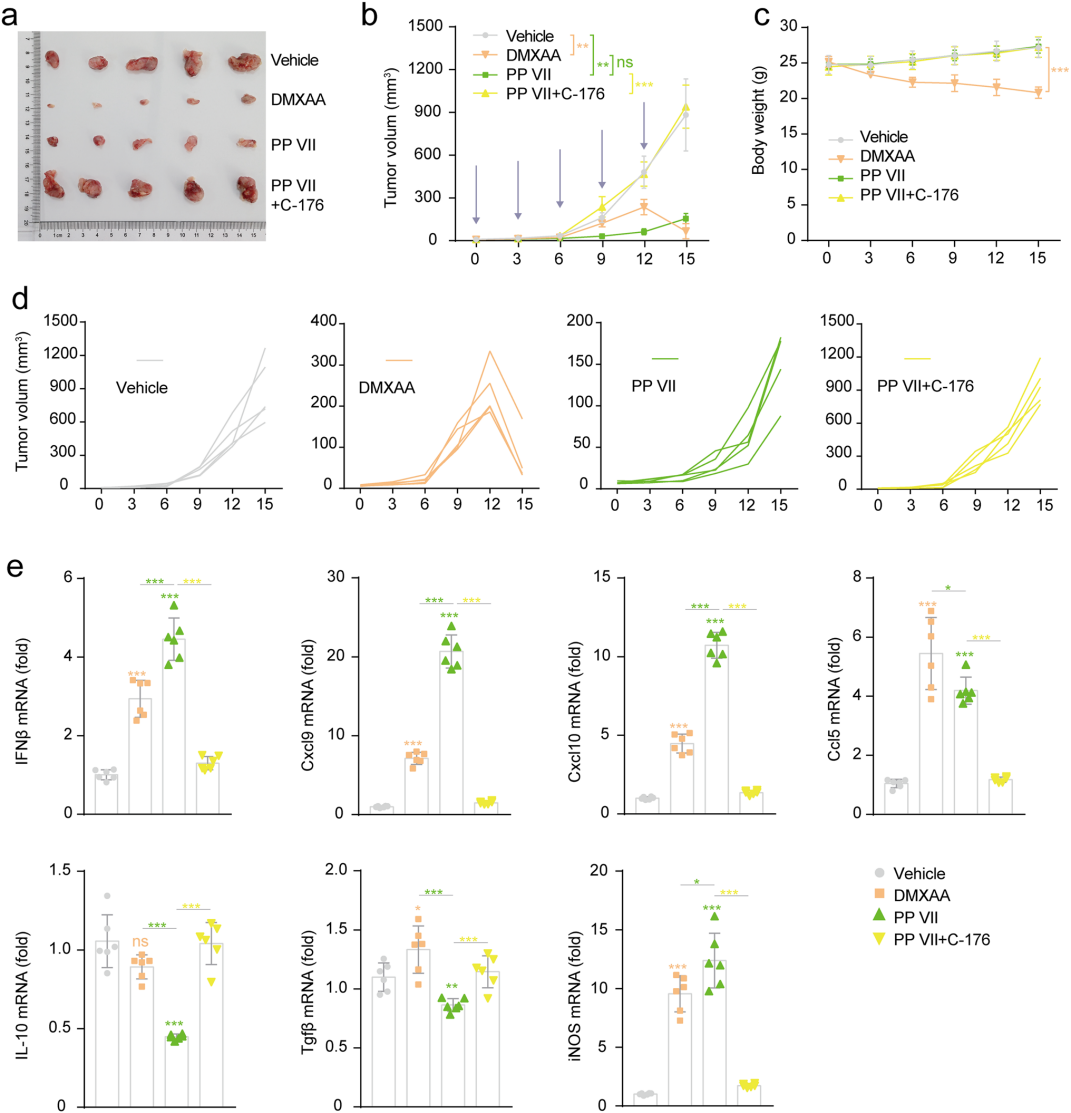
PP VII exerted anti-tumor efficacy dependent on STING activating while induces chemokines intratumorally. (**a**–**e**) C57BL/6 mice (n = 5 per group) with LLC tumors were injected i.p. with vehicle, DMXAA (15 mg/kg), PP VII (7.5 mg/kg), or the combination of PP VII and C-176 (750 nM per mouse) five times (indicated by bule arrows in (**b**). Gross appearance of the tumors extracted from tumor-bearing mice at day 15 after treatment initiation (**a**). Tumor volumes were measured at indicated time points (**b**) and the individual tumor volumes for each treatment are also presented (**d**). Mice were weighted as indicated (**c**). Tissue sections from LLCs tumors received indicated treatments were analyzed by qPCR (**e**). Data are depicted as mean + SD of six samples from pools of two mice. ***p < 0.001, **p < 0.01, *p < 0.05, *ns* no significant.

To aimed to evaluate the efficacy of PP VII in host defense against tumor growth, LLCs were subcutaneously transplanted into immunocompetent C57BL/6 J mice. PP VII significantly induced tumors regression Compared with vehicle in a stable and durable manner, but the lasting anti-tumor efficacy of PP VII was abolished by C-176. DMXAA showed modest tumor-suppressing effect at the early stage, however, the tumor shrank surprisingly with the accumulation of administrations (Fig. 8a,b,d). Injections with PP VII for 2 weeks did not show any weight loss, indicating that PP VII was well tolerance in mice. Conversely, DMXAA resulted in weight loss from beginning to end (Fig. 8c).

Then, we evaluated the proinflammatory genes associated with STING signaling and T cell infiltration on tumor tissues and found the mRNA level of Ifnβ, Cxcl9, Cxcl10, Ccl5 and iNOS was significantly enhanced by PP VII or DMXAA challenge, We also found a reduction of anti-inflammatory genes IL-10 and TGF-β in mice challenged by PP VII, but not obvious by DMXAA, this role of PP VII in regulating inflammatory response in TME was rejected when mice were simultaneously injected with C-176 (Fig. 8e).

**Figure 9:**
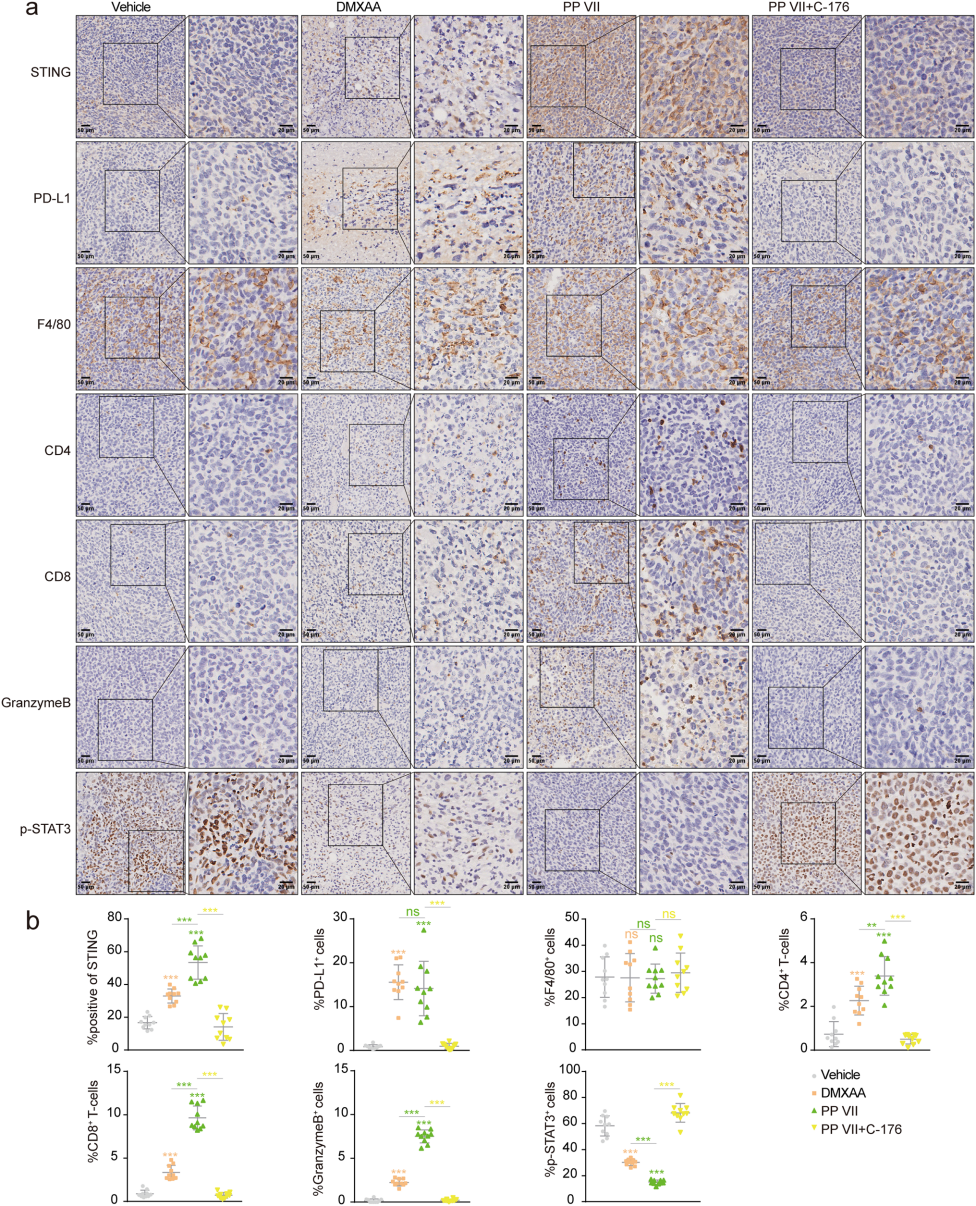
PP VII induces CD8^+^ T cell infiltration and inactivates STAT3 transcription intratumorally dependent on STING. (**a**,**b**) IHC analysis (**b**) for indicated tumors is depicted as mean + SD of ten random fields in 3 samples from pools of 3 mice showing one representative image (**a**). Scale bars denote 20 or 50 µm. ***p < 0.001, **p < 0.01, *p < 0.05, *ns* no significant.

We further assessed STING and downstream PD-L1 and p-Stat3, as well as tumor-infiltrating immune cells by performing IHC. Since STING was activated drastically under PP VII challenge, the accumulation of CD8^+^ T-cells and Granzyme B^+^ cells dramatically deepened, beyond that of DMXAA. Given that Granzyme B is the specific indicator of killer cytotoxic T lymphocytes, our data indicated that the chemotaxis of Granzyme B^+^ CD8^+^ T cells were vastly strengthened by PP VII, but that was disrupted when mice were simultaneously subjected to C-176. The recruitment of CD4^+^ T cells was also increased in PP VII and DMXAA injected mice, suggesting an elevated antigen-presentation capability. Macrophages were indeed heavily infiltrated in all tumor tissues, no change was detected in each treatment for F4/80, the major indicator of murine macrophages^15^, pointing that PP VII regulates macrophages transformation rather than induces their accumulation. Additionally, the induction of PD-L1 and the reduction of p-Stat3 under PP VII challenge was disturbed by C-176 (Fig. 9a,b). Collectively, our data indicate that PP VII boosts proinflammatory chemokines secretion to recruit Granzyme B positive CD8^+^ T lymphocytes for intratumoral infiltration thus exerting anti-tumor immunity, as well as negatively regulates Stat3 activity to suppress downstream immunosuppressive cytokines, accompanied with PD-L1 elevation, all that are determined by STING and probably associated with macrophages.

## Discussion

Mounting evidence suggested that STING is involved in effective antitumor adaptive immunity. Macrophages, accounting for the majority of tumor-infiltrating immune cells, exert multiple effects in antigen presentation, inflammatory responses triggering, and T cells chemotaxis ^10,12–14,16^. Here we illustrated how macrophages bridge the innate and acquired immune systems to trigger anti-tumor responses. Our data showed that STING-activated macrophages release massive cytokines and chemokines in vitro, meanwhile, promote cytotoxic T cell infiltration and enhance the killing activity of them in local TIME in vivo. Furthermore, we found polyphyllin VII, a natural small molecule compound, primes macrophage to M1 type governed by STING, and exert anti-tumor efficacy upon Granzyme positive CD8^+^ T cells accumulation.

Our studies also help to elucidate the mechanism of PD-L1 induction on host cells and verified that targeting STING by PP VII and DMXAA drives a pronounced PD-L1 level both in macrophages cell lines and tumor tissues of immunocompetent mice. Given that the promotion of M1 macrophage polarization by PP VII comes together with induction of PD-L1, both of which can be initiated by IFN-γ, excluding the LPS/TLR4 cascade, we speculate the effect of PP VII is associated with an unknown interaction between STING and PD-L1. Imaging that once STING is triggered, hitting the accelerator to the maximum, macrophages will initiatively prime to an immune-activated M1 type for fighting, followed by inevitably upregulate the immune checkpoint PD-L1, a brake to prevent excessive inflammatory response and cytokine storm. Since PD-L1 low-expression is the main reason for the low response rate of PD-L1 blockade^49,50^, our data provide a scientific rational for combining PP VII with anti-PD-L1 therapy to overcome the resistance, further studies will be necessary to interrogate the contribution.

It is generally accepted that TAMs are close to M2 macrophages induced by Th2-type cytokine IL-4^10,15,16^. But our data showed that the Stat3-activated macrophages induced by IL-6 are closer to TAMs at the levels of promoting production of immunosuppressive factors and leading to malignant transformation of tumor cells. Consistent with our results, persistently activated STAT3 in both host cells and tumor cells suppresses the inflammation response^51–53^ and promotes the transformation of immunosuppressive immune-cells^28^. Our data provide evidence for elucidating the leading role of STING in regulating STAT3-propagated crosstalk between immune cells and tumor cells. Admittedly, the direct molecular interaction in this process needs to be further investigation.

Additionally, we detected no significant change of IL-10 and TGF-β expression in IL-4 induced macrophages, neither at mRNA nor protein level (data not shown). Interestingly, PP VII inhibits the protein expression of IL-4-induced CD206 in RAW264.7s, without affecting Stat6. We have no further study on this model, but given the results of other researchers, this reduction probably is due to STING excited by PP VII^54,55^.

As agonists, PP VII and DMXAA have their own patterns of action. PP VII is effective in both murine macrophage cell line RAW264.7 and differentiated human macrophage cell line THP-1 as we showed. And DMXAA had been confirmed to be mouse-specific. PP VII continuously regressed tumor growth, while DMXAA took effect at the late stage. Moreover, the CD8^+^ T cells infiltration and the Granzyme B activity in DMXAA-treated tumors were not as strong as those treated by PP VII. However, the final tumor volume of DMXAA-treated group was smaller, although there was no significance in the difference. To find out the reason, we observed removed tumor tissues and found that the color of tumors treated by DMXAA was apparently whitish and hardly detected blood vessels, while those accepted other treatments were rich in vessels filling blood. To the best of our knowledge, DMXAA is initially applied as a vascular disrupting agent, coinciding with tumors with abundant blood supply formed by LLCs subcutaneous inoculated in C57BL/6 J mice, the role of DMXAA in vessels disruption contributes to tumor suppression as well^56^. Our data showed that PP VII drives a much heavier infiltration of Granzyme B positive cytotoxic T lymphocytes upon boosting proinflammatory chemokines. We have confirmed that this boosted secretion of proinflammatory chemokines is attributed to macrophage priming in vitro, but this role of macrophages in vivo needs further confirmation.

Given the fact that macrophages serve as medium for communication between host and pathogen, their inflammatory response form an inescapable part for shaping the immune environment. In summary, our data show that PP VII resulted in alteration of gene expression programs in macrophage transformation to enhance anti-tumor immunity and restrain immunosuppression via STING-dependent type I IFN and STAT3 signaling. Moreover, PP VII may benefit immune-checkpoint blockade therapy, since it elevates PD-L1 expression on macrophages. Considered that STING signaling is also the major innate antivirus pathway by managing type I IFN production^57^, our study suggests a potential application of PP VII in anti-tumor and antivirus immunotherapy.

## Methods

### Cell culture

Cell lines RAW264.7, THP-1, A549, LLC were obtained from the cell bank of the Chinese academy of science (Shanghai, China). The cells were verified with STR profiling and identification without mycoplasma (http://www.cellbank.org.cn/). They were regularly maintained by Plasmocin prophylactic (a removal agent to prevent mycoplasma contamination, invivoGen). RAW 264.7s, LLCs, A549s were cultured in Dulbecco’s modified Eagle’s medium (DMEM) (Corning, US). THP-1s were cultured in β-mercaptoethanol (21985-023, Gibico)-containing RPMI1640 medium (Corning, US). Growth medium were supplemented with 10%(v/v) of heat inactivated Fetal Bovine Serum (FBS) (10099141, Giboco), and penicillin/streptomycin (100U/ml) (15140163, Gibico). All cell lines were cultured at 37° in a humidified incubator supplied with 5%(v/v) CO_2_.

### Cell stimulation

THP-1 cells were differentiated with 100 ng/ml PMA (P1585,Sigma) for 24 h then treated with 100 ng/ml IFN-γ (300-02, PeproTect), or 50 ng/ml IL-6 (200-06, PeproTect), otherwise 20 ng/ml IL-4 (200-04, PeproTect) for another 24 h. RAW264.7s were incubated with 100 ng/ml IFN-γ (315-05, PeproTect), or 50 ng/ml IL-6 (216-16, PeproTect), or 20 ng/ml IL-4 (214-14, PeproTect), or 1 µg/ml LPS (L4524, Sigma)for 24 h. RAW264.7s and THP-1s were incubated with medium containing 1% (v/v) FBS for 24 h, after stimulation as indicated. Cell supernatants were harvested as conditional medium (CM) by centrifugation at 1500* g* for 5 min to remove all the debris. If not otherwise indicated, small molecule compounds, inhibitors, agonists were added to the cells 2 h before stimulation using the following concentrations: 2 µm PP (CAS No.76296-75-8, Apexbio, Houston) for RAW264.7 and 1 µm PP for THP-1, 2 µm C-176 (S6575, Selleck) and 50 µg/ml DMXAA (S1537, Selleck) for RAW264.7, 1 µm H-151 for THP-1 (inh-h151, invivoGen).

### Cell viability

THP-1 and RAW264.7 cells were respectively seed at 25,000 cells or 6000 cells per well in 96 well plates. THP-1s were differentiated into macrophages as described before treated with PP at different concentrations. A549 and LLC cells were seeded at 3000 cells/well in 96 well plates and respectively incubated with CM derived from THP-1s or RAW264.7s. Cells were counted every 24 h by a cell counting kit-8 (cck-8) agent (Dojindo) according to the manufacturer’s instruction.

### Flow cytometry

For cell death assay, RAW264.7s, THP-1s, LLCs, A549s was seeded into 6-well plates and treated with PP VII or different CMs for 24 h followed by harvested for staining. Apoptosis Detection Kit (556547, BD Biosciences) was performed as manufacturer’s recommendation. Cells were incubated with FITC-Annexin V in a buffer containing propidium iodide (PI) then processed by FACSVerse (BD Biosciences). For RAW264.7s staining, single-cell suspensions were prepared and stained according to standard protocol with Brilliant Violet 421 anti-mouse CD86 (105031, Biolegend) and APC anti-mouse F4/80 (123116, Biolegend). The data were acquired on a Fortessa Platform (BD Biosciences) and analyzed with FlowJo software (Version V10.0.7, Tree Star).

### SiRNA transfection

For small interfering RNA (siRNA) knockdown in RAW264.7s, cells were transfected with STING specific siRNAs (si-mSTING_1: 5′-AGAGG TCACCGCTCCAAATAT-3′, si-mSTING_2: 5′-ATGATTCTACTATC GTCTTAT-3′, si-mSTING_3: 5′-C AACATTCGATTCCGAGATAT-3′) and IRF3 specific siRNAs (si-mIRF3_1: 5′-CCAATGTGAACAACTTCCTAA-3′, si-mIRF3_2: 5′-CCCACG CTACACTCTGTGGTT-3′, si-mIRF3_3: 5′-CGA AGTTATTTGATGGCCTGA-3′) or scrambled siRNA controls (50 nM) using Lipofectamine RNAiMax (13778075, Life technologies). Fifty hours after transfection, PP was added into the medium, 2 h later, cytokines were added as indicated.

### Immunoblotting

Cells were lysed in 3×Laemmli buffer, denatured for 10 min at 95° and separated by Tris–glycine denaturing SDS-PAGE. Proteins were blotted onto polyvinylidene fluoride membranes, blocked in 5% milk and incubated with indicated primary and corresponding secondary antibodies. Chemiluminescent signals were recorded with a CCD-camera and data were analyzed with Image J software. The antibodies were as follows: Rabbit anti-STAT1 (1:1000, 9172), anti-phospho-STAT1 (1:1000, 9167/Tyr701), anti-STAT3 (1:1000, 9139), anti-phospho-STAT3 (1:1000, 9145/Tyr705), anti-STAT6 (1:1000, 5397), anti-phospho-STAT6 (1:1000, 56554/Tyr641), anti-STING (1:1000, 13,647), anti-TBK1 (1:1000, 3504), anti-phospho-TBK1 (1:1000, 5483/Ser172), anti-IRF3(1:1000, ab68481), anti-phospho-IRF3(1:1000, 4947/Ser396), PCNA (1:1000, 13,110), E-cadherin (1:1000, 3195), Vimentin (1:1000, 5741), α/β Tubulin (1:1000, 2148), βActin (1:1000, 4970), and antibody against murine cGAS (1:1000, 31,659), human cGAS (1:1000, 15,102), human phospho-STING (1:1000, 19,781/Ser366), were purchased from Cell Signaling. Anti-murine PD-L1 (1:1000, ab213480), human PD-L1 (1:1000, ab213524), iNOS (1:1000, ab178945), MRC1 (1:1000, ab64693), GAPDH (1:5000, ab181602), HRP-conjugated secondary antibody (1:5000, ab6721) were from Abcam. The band intensity of proteins was quantified by using ImageJ software (National Institutes of Health, Bethesda, MD, USA), and the relative expression of protein to β-actin/GAPDH/Tubulin was normalized and obtained.

### Immunostaining

RAW264.7 cells were seeded on coverslips and stimulated as indicated, fixed with 4% (v/v) paraformaldehyde, permeabilized with Triton X-100 in PBS, blocked with 3% BSA before incubation with primary antibodies followed by Alexa Flour secondary antibody. Nuclei were stained with DAPI in mounting medium (Beyotime). Fluorescence images were taken with a Leica SP8 confocal laser scanning microscope and processed in Image J software. The antibodies used were rabbit anti-STING (19851-1-AP, Proteintech), anti-iNOS (ab178945, Abcam), and Alexa Flour 488 (ab150077, Abcam).

### Quantitative RT-qPCR

Total RNA was isolated using Trizol (Invitrogen) and the cDNA were synthesized using the Prime Script RT reagent Kit (RR047A, Takara). Quantitative RT-qPCR was performed in duplicates using TB Green Premix Ex Taq (RR420A, Takara) on Step One Plus Real-Time PCR System (Thermo). GAPDH was used as an endogenous normalization control to obtain relative expression data. The details of the primers are given in supplementary file (Supplementary material1_primers sequences).

### Enzyme-linked immunosorbent assay

Protein levels of secreted cytokines and chemokines in CM were detected by ELISA according to the manufacturer’s instructions. The ELISA Kit used were: mouse IFN beta Kit (ab252363, Abcam), Mouse CXCL10 Kit (ab214563, Abcam), Human CXCL10 Kit (ab173194, Abcam), Mouse CXCL9 Kit (ab100725, Abcam), Human CXCL9 Kit (ab100595, Abcam). Murine IL-10 Kit (1211002, DAKEWE), human IL-10 Kit (1111002, DAKEWE) and murine TGF-β1 Kit (1217102, DAKEWE), human TGF-β1 Kit (1117102, DAKEWE).

### Transwell

Cancer cell invasion was performed in a transwell chamber. The tumor cell suspension (1×10^5^ cells in serum-free medium) was placed in the upper chamber. The lower compartment filled 0.6 ml macrophage CM with 20% FBS. Cells were allowed to transfer within 24 h at 37°, then fixed with methanol and stained with crystal violet . The upper chamber was pre-coated with a layer of Matrigel (Corning). Staining cells were photographed and counted in 5 random fields.

### Animals

Male C57BL/6 mice (7 weeks old) were generated by Shanghai Slac Laboratory, and maintained under specific pathogen-free conditions. All animal experiments were conducted in according with relevant guidelines. The study was officially approved by Institutional Animal Care and Use Committee at Shanghai University of Traditional Chinese Medicine (approval numbers PZSHUTCM190920001).

### Tumor growth and treatment

5×10^5^ LLC cells in 100 µl PBS were subcutaneously injected into the flank of mice. Four days after tumor inoculation, mice were separated into treatment groups to ensure all cohorts had comparable tumor volume. Treatment was initiated and continued until tumors reached 20 mm in any direction. DMSO-reconstituted PP diluted in PBS (Corning) injected intraperitoneally at a dose of 7.5 mg/kg, alone or immediately after C-176 (750 nM per mouse) injection. DMXAA were administered i.p. at 15 mg/kg. These treatments repeated five times with 3-d intervals. Tumors were measured with a digital caliper by length (a) and width (b) and calculated as tumor volume = ab^2^/2.

### Immunohistochemistry

Tumor tissues were fixed in 4% paraformaldehyde, embedded in paraffin, cut into sections and stained according to standard procedures. The antibodies used were: anti-F4/80 (ab111101, Abcam), anti-PD-L1 (ab205921, Abcam), anti-CD4 (ab183685, Abcam), anti-CD8 (ab209775, Abcam), anti-Granzyme B (ab4059, Abcam), anti-STING (13647, Cell signaling), anti-phospho-Stat3 (9145, Cell Signaling). IHC analysis of STING was performed by IHC profiler in image J. Quantitative results of F4/80, CD4, CD8, Granzyme B, p-Stat3, PD-L1 were determined by manual counting of positive cells and total cells in 10 fields of 3 tumors.

### Statistical analysis

No statistical method was used to predetermined sample size. Sample sizes were instead determined on the basis of previous experimental experiences or based on general practice in the field. Replicates are biological replicates. Mice were randomly allocated to groups. For histological analysis of tissues, investigators were blinded to the experiment conditions. Data were analyzed by Prism 7.0 software (GraphPad) and presented as mean ± SEM or SD. The *p* values were assessed using two-tailed unpaired Student’s t test with *p* values considered significant as follows: *P < 0.05, **P < 0.01, ***P < 0.001.

## Supplementary information

Supplementary Information 1.
