# Supplementary material for: Targeting macrophage priming by polyphyllin VII triggers anti-tumor immunity via STING-governed cytotoxic T-cell infiltration in lung cancer
Source: Sci Rep. 2020 Dec 7;10:21360. doi: 10.1038/s41598-020-77800-w (PMC7721813; doi:10.1038/s41598-020-77800-w)
Supplement: Supplementary file 1 — Supplementary Information 1. [file 41598_2020_77800_MOESM1_ESM.pdf]

Targeting macrophage priming by Polyphyllin VII triggers anti-tumor immunity via STING-governed cytotoxic T-cell infiltration in lung cancer

Jinglu Yu [a, b], Haibin Deng [a, b, *] and Zhenye Xu [a, b, *]

[a] Department of oncology, Shanghai University of Traditional Chinese Medicine Longhua Hospital, Shanghai 200032, China

[b] Shanghai University of Traditional Chinese Medicine, Shanghai 200030, China

* correspondence should be addressed to H. D. (HaibinDeng@126.com) ; Z. X. (Zhenye_Xu@126.com)

Supplementary material1_primers sequences

Gene	Sense	Antisense
mmGapdh	CATCACTGCCACCCAGAAGACTG	ATGCCAGTGAGCTTCCCGTTCAG
mmIfnb1	GCCTTTGCCATCCAAGAGATGC	ACACTGTCTGCTGGTGGAGTTC
mmCcl5	CTGCTGCTTTGCCTACCTCT	TCCTTCGAGTGACAAACACG
mmCxcl9	CGAGGCACGATCCACTACAA	AGGCAGGTTTGATCTCCGTT
mmCxcl10	AGTGCTGCCGTCATTTTCTG	TCCCTATGGCCCTCATTCTCA
mmCiita	ACCTTCGTCAGACTGGCGTTGA	GCCATTGTATCACTCAAGGAGGC
mmCd86	ACGTATTGGAAGGAGATTACAGCT	TCTGTCAGCGTTACTATCCCGC
mmCd40	ACCAGCAAGGATTGCGAGGCAT	GGATGACAGACGGTATCAGTGG
mmPdl1	TGCGGACTACAAGCGAATCACG	CTCAGCTTCTGGATAACCCTCG
mmiNOS	GAGACAGGGAAGTCTGAAGCAC	CCAGCAGTAGTTGCTCCTCTTC
mmTnfa	GGTGCCTATGTCTCAGCCTCTT	GCCATAGAACTGATGAGAGGGAG
mmIrf3	CGGAAAGAAGTGTTGCGGTTAGC	CAGGCTGCTTTTGCCATTGGTG
mmIrf7	CCTCTGCTTTCTAGTGATGCCG	CGTAAACACGGTCTTGCTCCTG
mmIrf1	TCCAAGTCCAGCCGAGACACTA	ACTGCTGTGGTCATCAGGTAGG
mmIrf5	CCTACAGAACCACTCTTGCCTG	CCTTGTGGGTTGCTGATGGTGA
mmIl-10	CGGGAAGACAATAACTGCACCC	CGGTTAGCAGTATGTTGTCCAGC
mmTgfb1	TGATACGCCTGAGTGGCTGTCT	CACAAGAGCAGTGAGCGCTGAA
hsGAPDH	GAGTCAACGGATTTGGTCGT	TTGATTTTGGAGGGATCTCG
hsIFNβ1	AGGACAGGATGAACTTTGAC	TGATAGACATTAGCCAGGAG

hsCXCL10	GGTGAGAAGAGATGTCTGAATCC	GTCCATCCTTGGAAGCACTGCA
hsCIITA	CTACTTCAGGCAGCAGAGGAGA	GCTGTGTCTTCCGAGGAACTTC
hsCD86	CCATCAGCTTGTCTGTTTCATTCC	GCTGTAATCCAAGGAATGTGGTC
hsPDL1	TGCCGACTACAAGCGAATTACTG	CTGCTTGTCCAGATGACTTCGG
hsIL-10	TCTCCGAGATGCCTTCAGCAGA	TCAGACAAGGCTTGGCAACCCA
hsTGFβ1	TACCTGAACCCGTGTTGCTCTC	GTTGCTGAGGTATCGCCAGGAA

Supplementary material2_full length gels and blots

Fig1.e

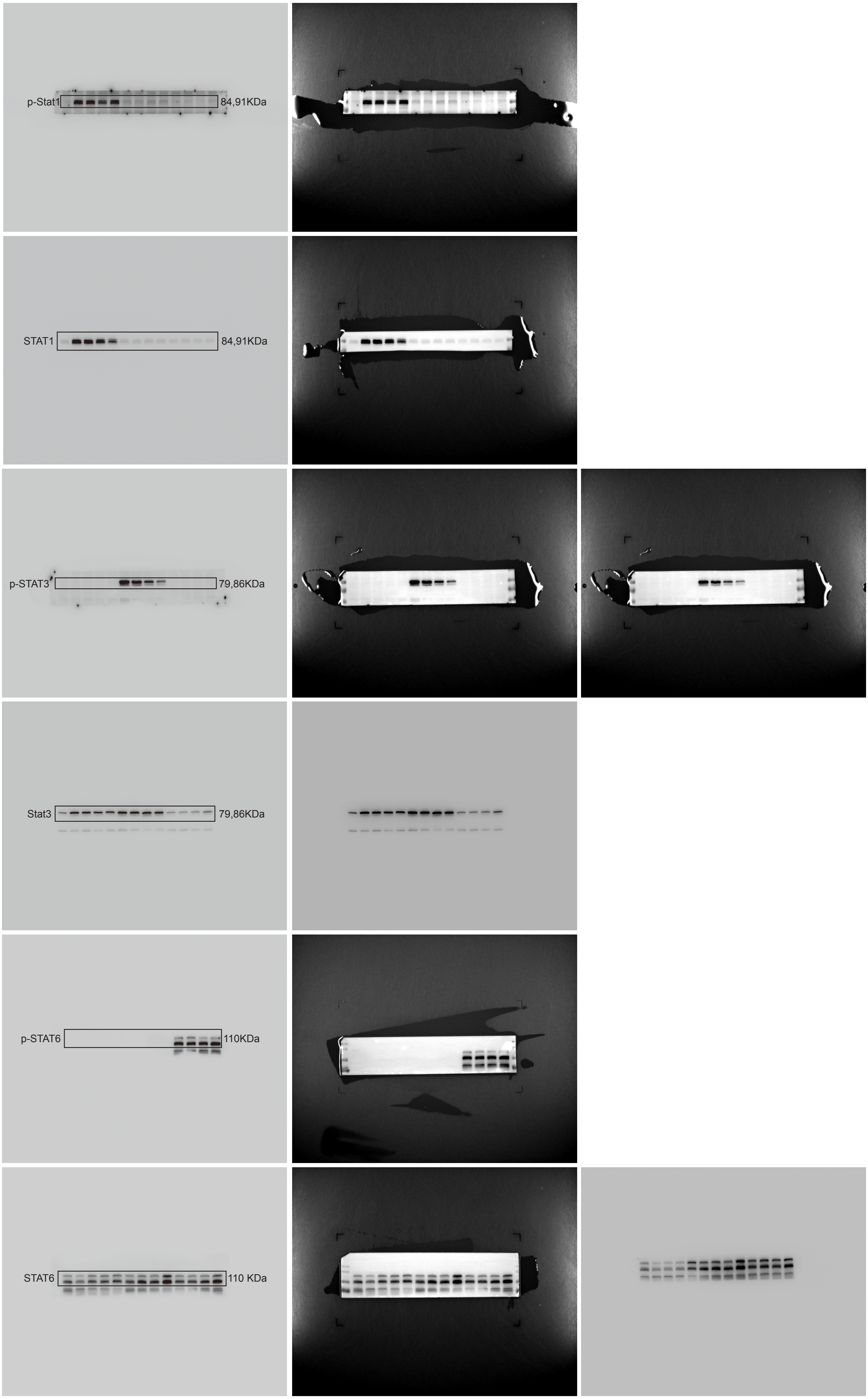

p-Stat1
84,91KDa
STAT1
84,91KDa
p-STAT3
79,86KDa
Stat3
79,86KDa
p-STAT6
110KDa
STAT6
110 KDa

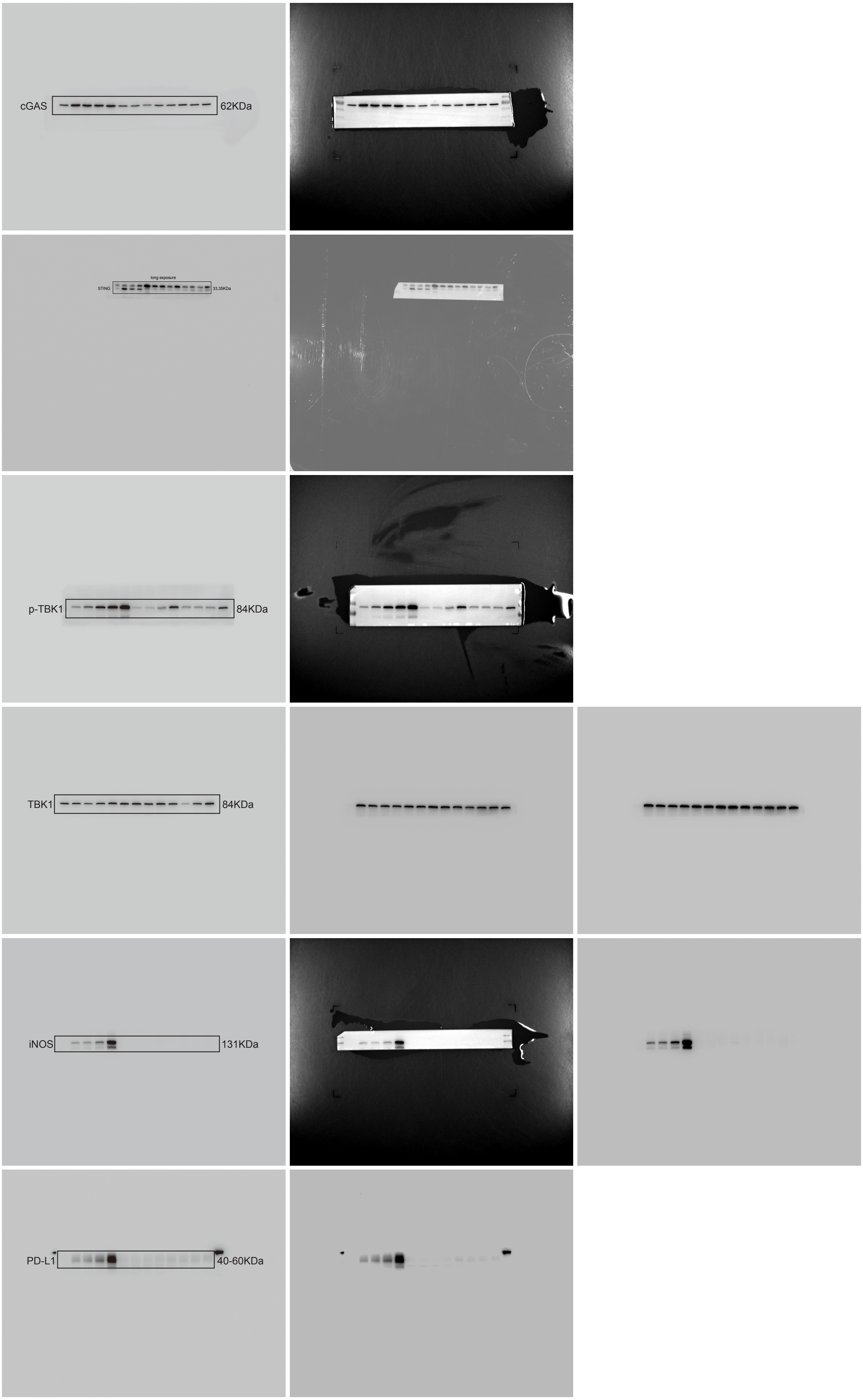
cGAS
62KDa
long exposure
STING
33,35KDa
p-TBK1
84KDa
TBK1
84KDa
iNOS
131KDa
PD-L1
40-60KDa

Fig1.f

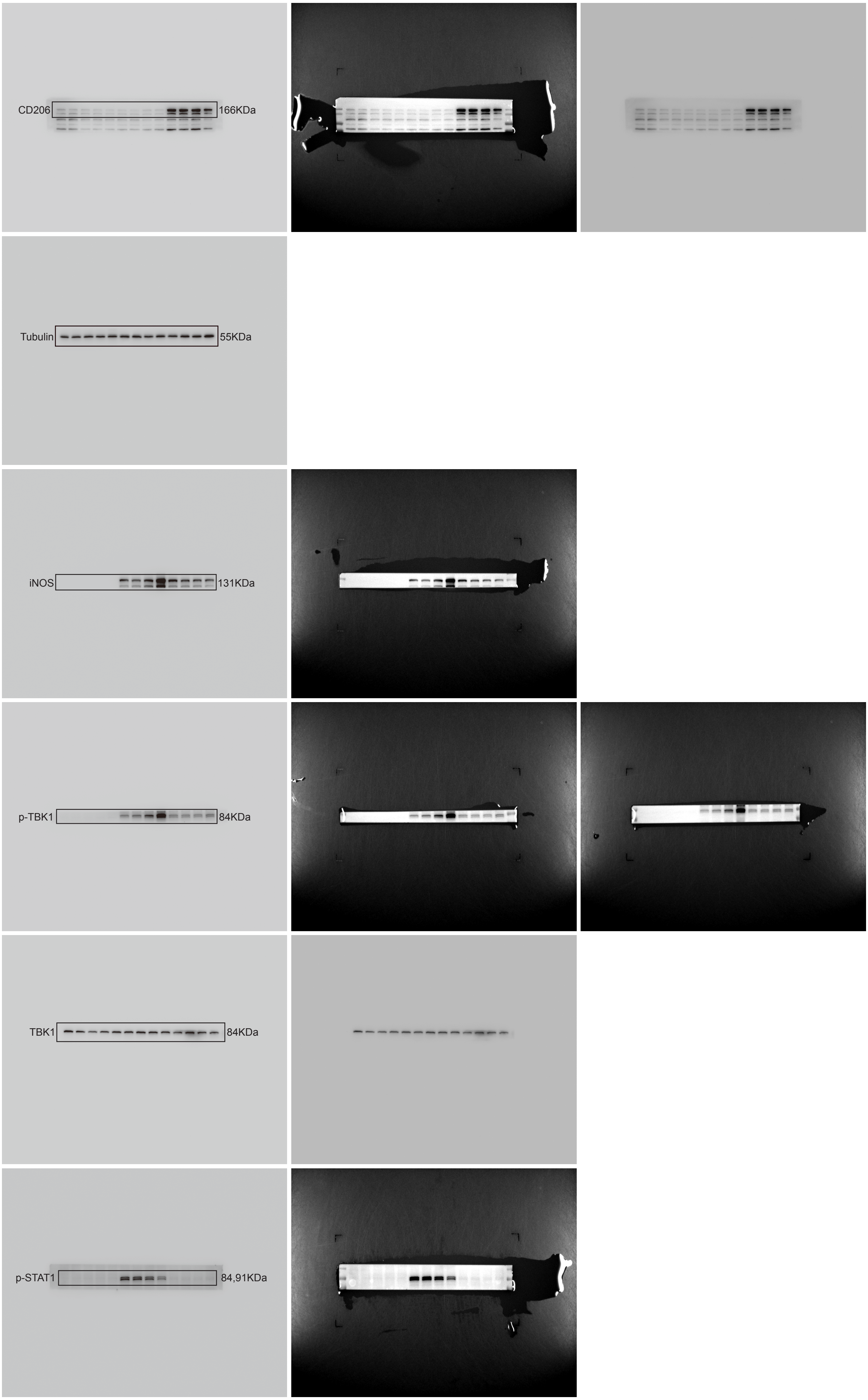
CD206
166KDa
Tubulin
55KDa
iNOS
131KDa
p-TBK1
84KDa
TBK1
84KDa
p-STAT1
84,91KDa

Fig1.g

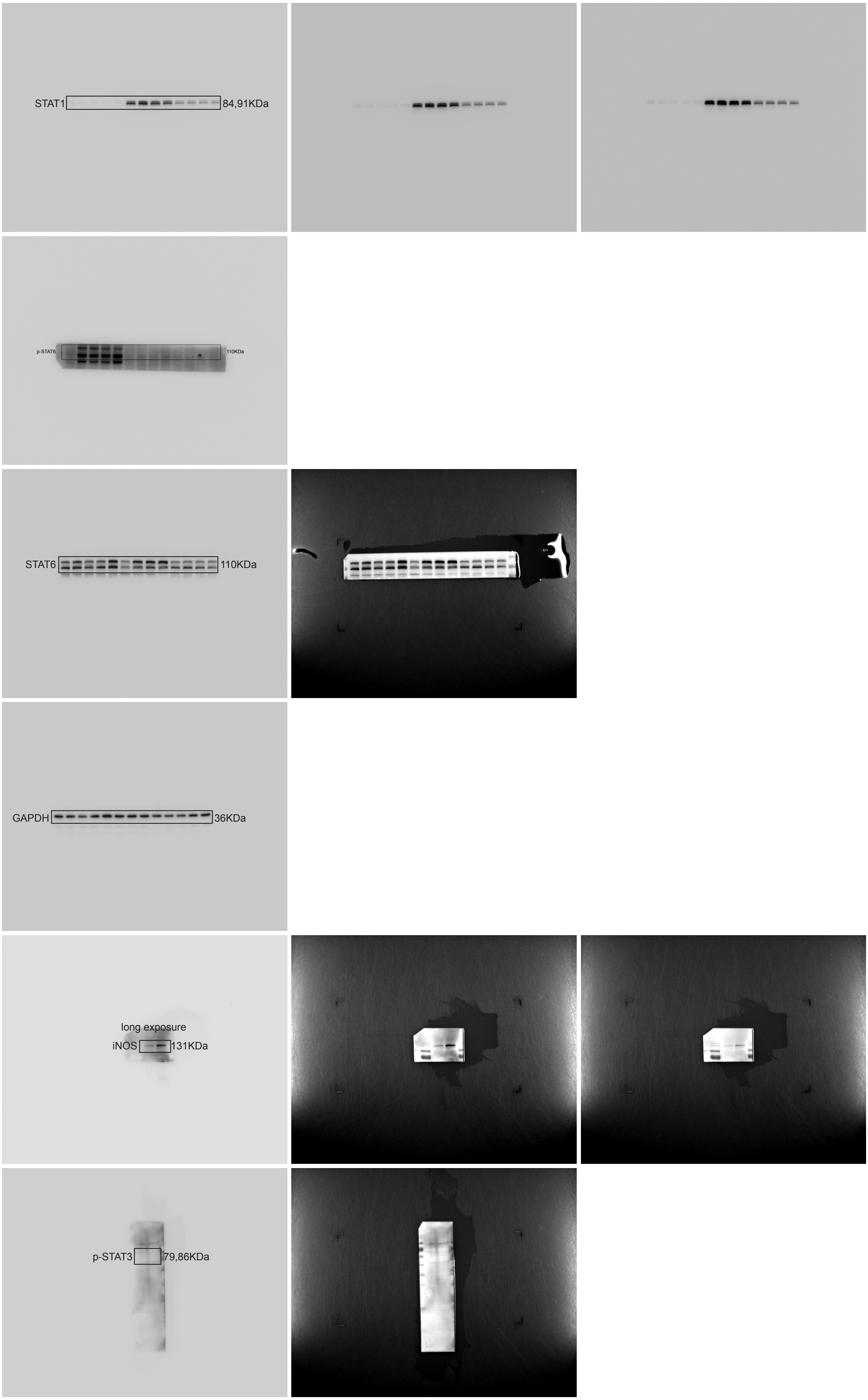
STAT1
84,91KDa
p-STAT6
110KDa
STAT6
110KDa
GAPDH
36KDa
long exposure
iNOS
131KDa
p-STAT3
79,86KDa

Fig1.h

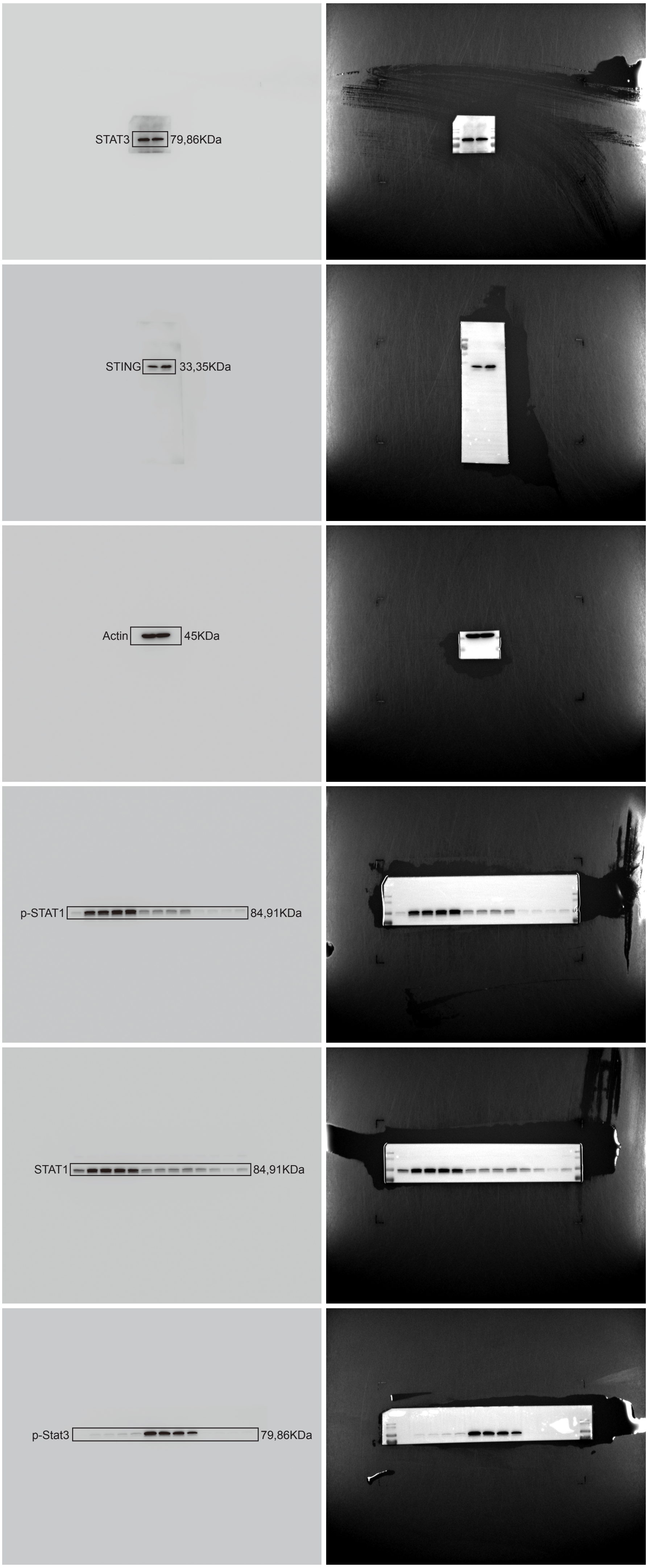
STAT3
79,86KDa
STING
33,35KDa
Actin
45KDa
p-STAT1
84,91KDa
STAT1
84,91KDa
p-Stat3
79,86KDa

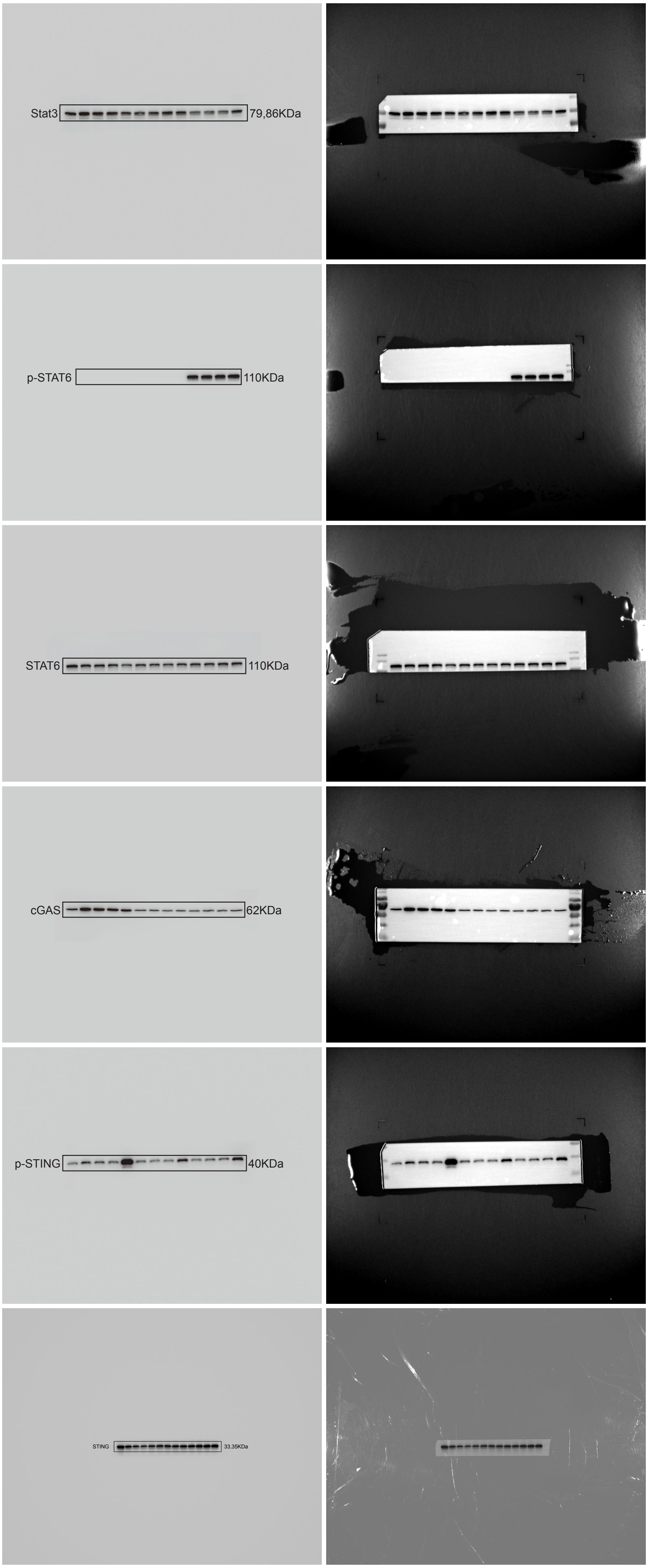
Stat3
79,86KDa
p-STAT6
110KDa
STAT6
110KDa
cGAS
62KDa
p-STING
40KDa
STING
33,35KDa

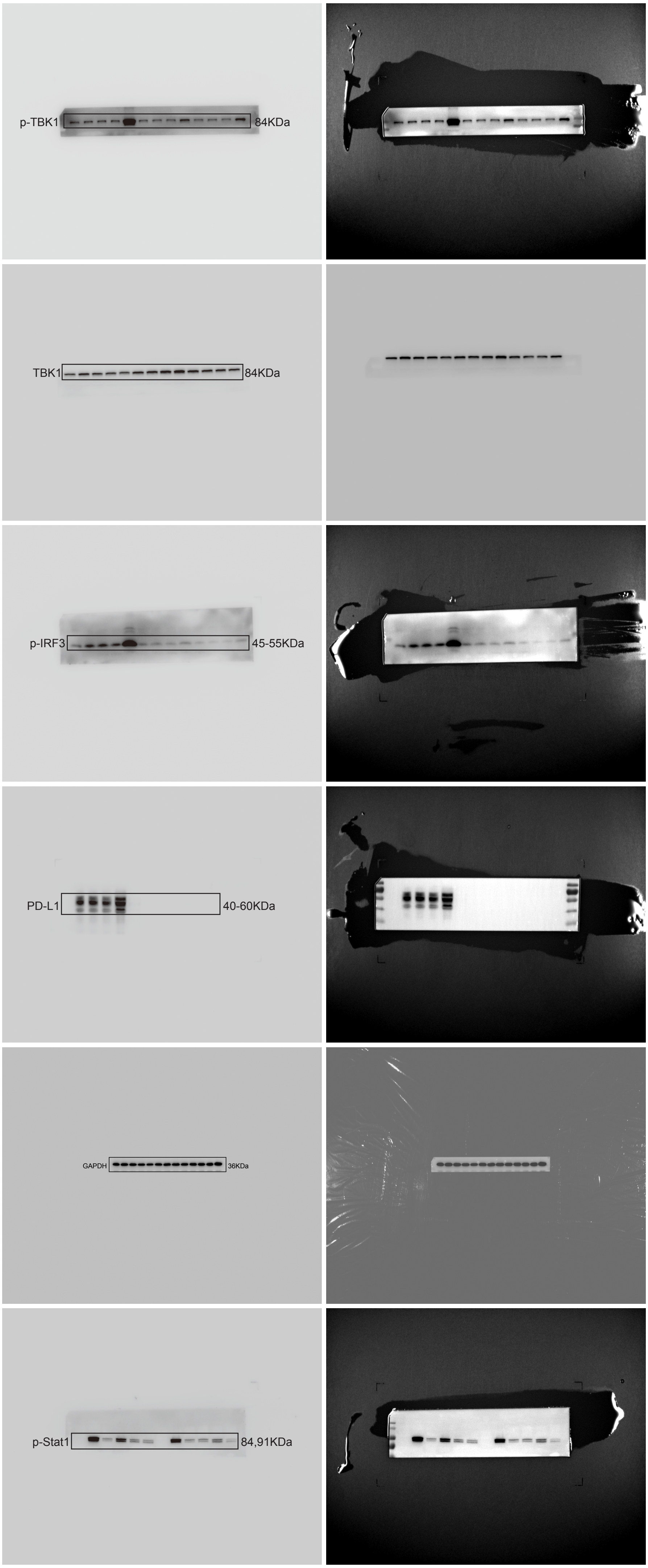
p-TBK1
84KDa
TBK1
84KDa
p-IRF3
45-55KDa
PD-L1
40-60KDa
GAPDH
36KDa
p-Stat1
84,91KDa

Fig2.a

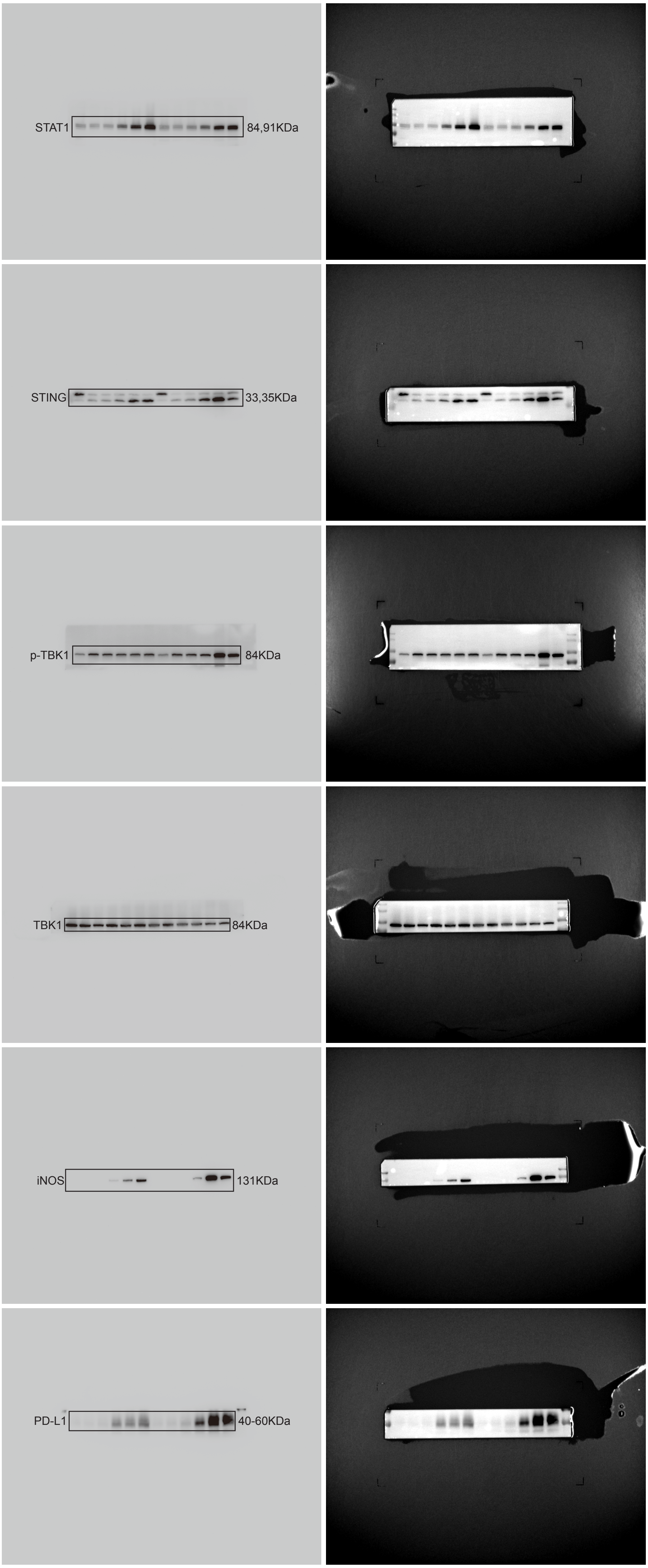

STAT1
84,91KDa
STING
33,35KDa
p-TBK1
84KDa
TBK1
84KDa
iNOS
131KDa
PD-L1
40-60KDa

Fig.2b

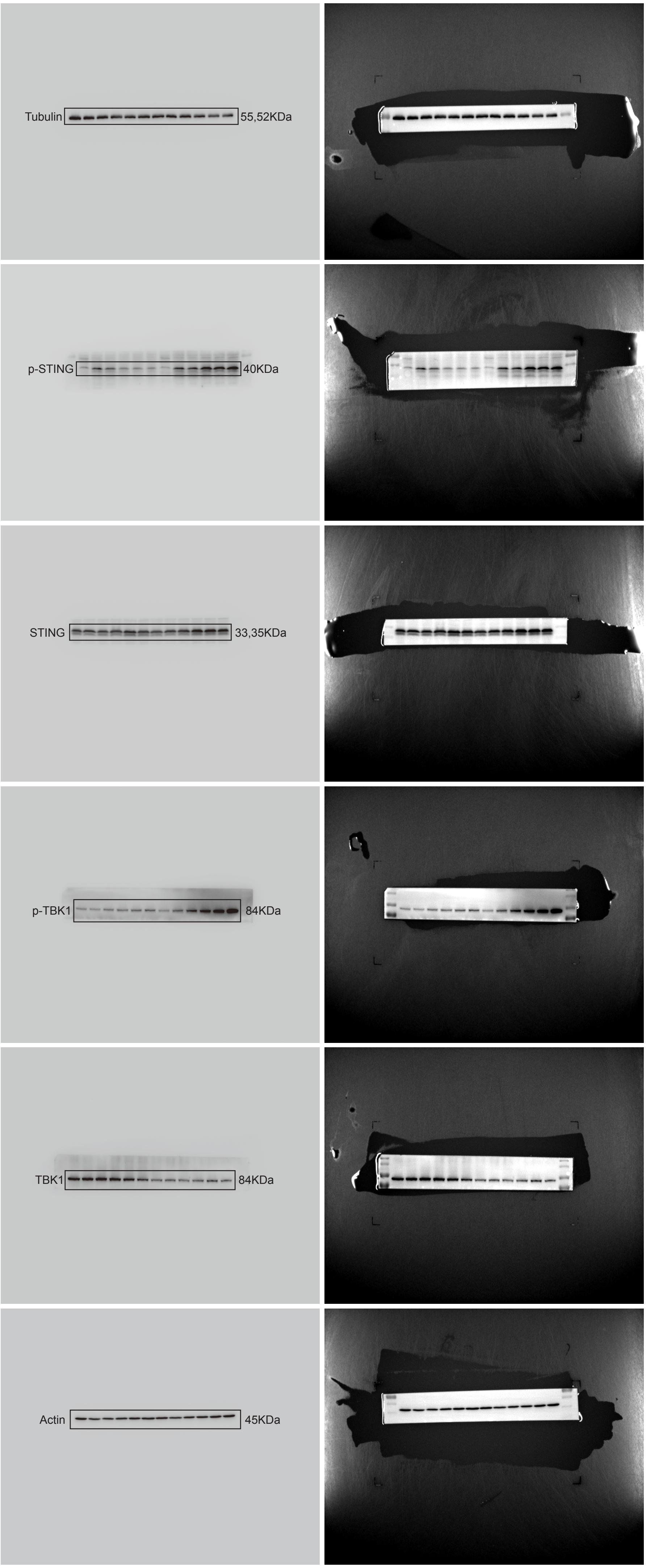
Tubulin
55,52KDa
p-STING
40KDa
STING
33,35KDa
p-TBK1
84KDa
TBK1
84KDa
Actin
45KDa

Fig2.c

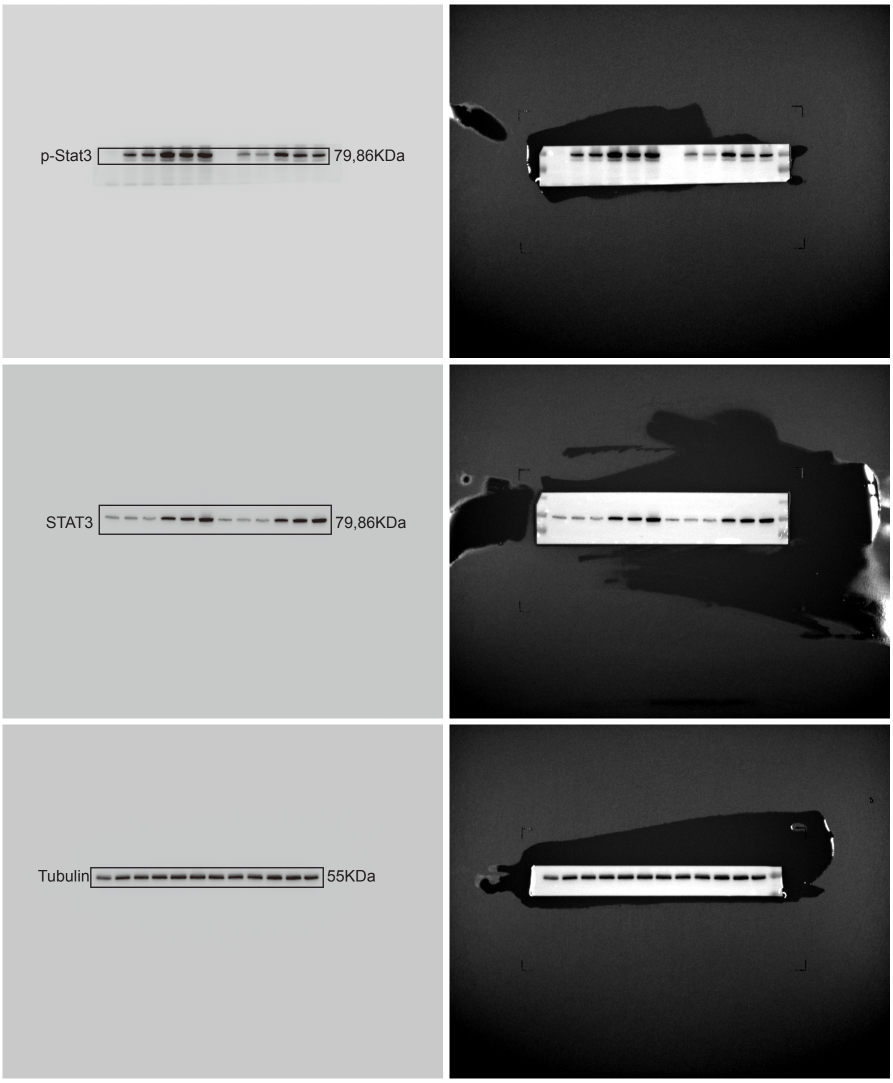
p-Stat3
79,86KDa
STAT3
79,86KDa
Tubulin
55KDa

Fig2.d

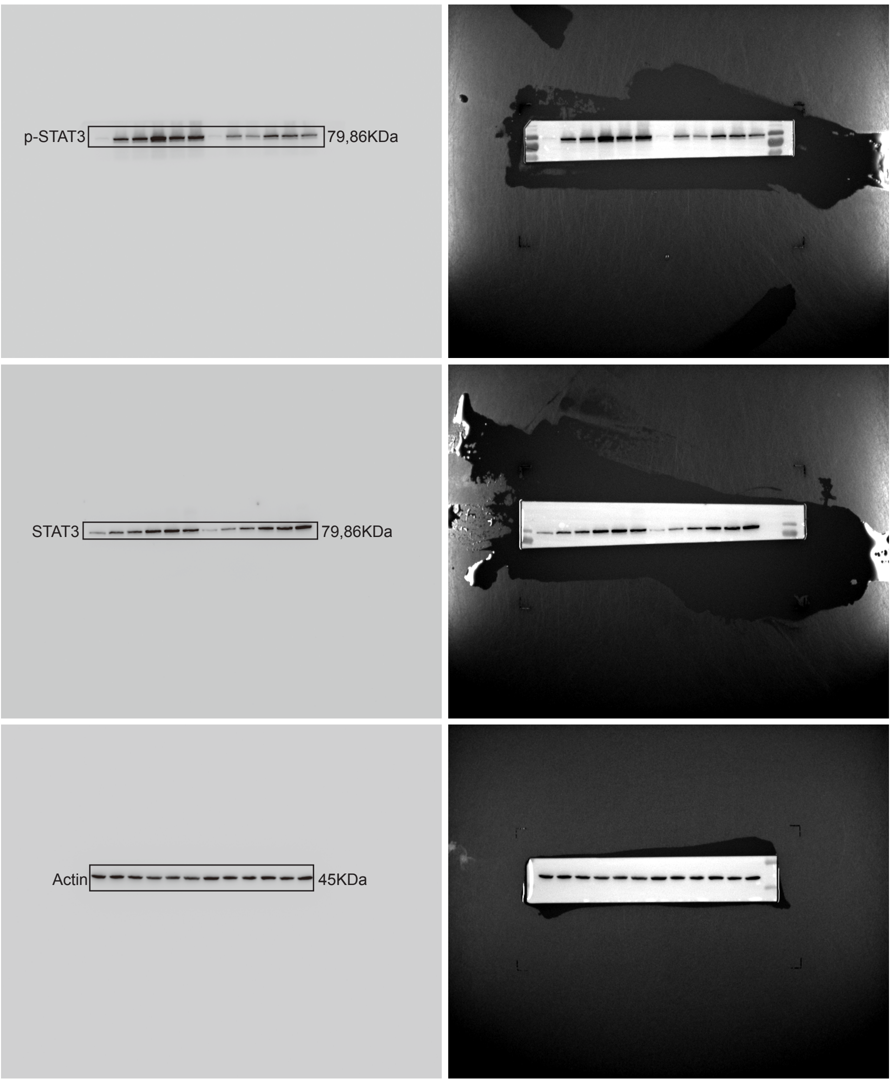
p-STAT3
79,86KDa
STAT3
79,86KDa
Actin
45KDa

Fig3.a

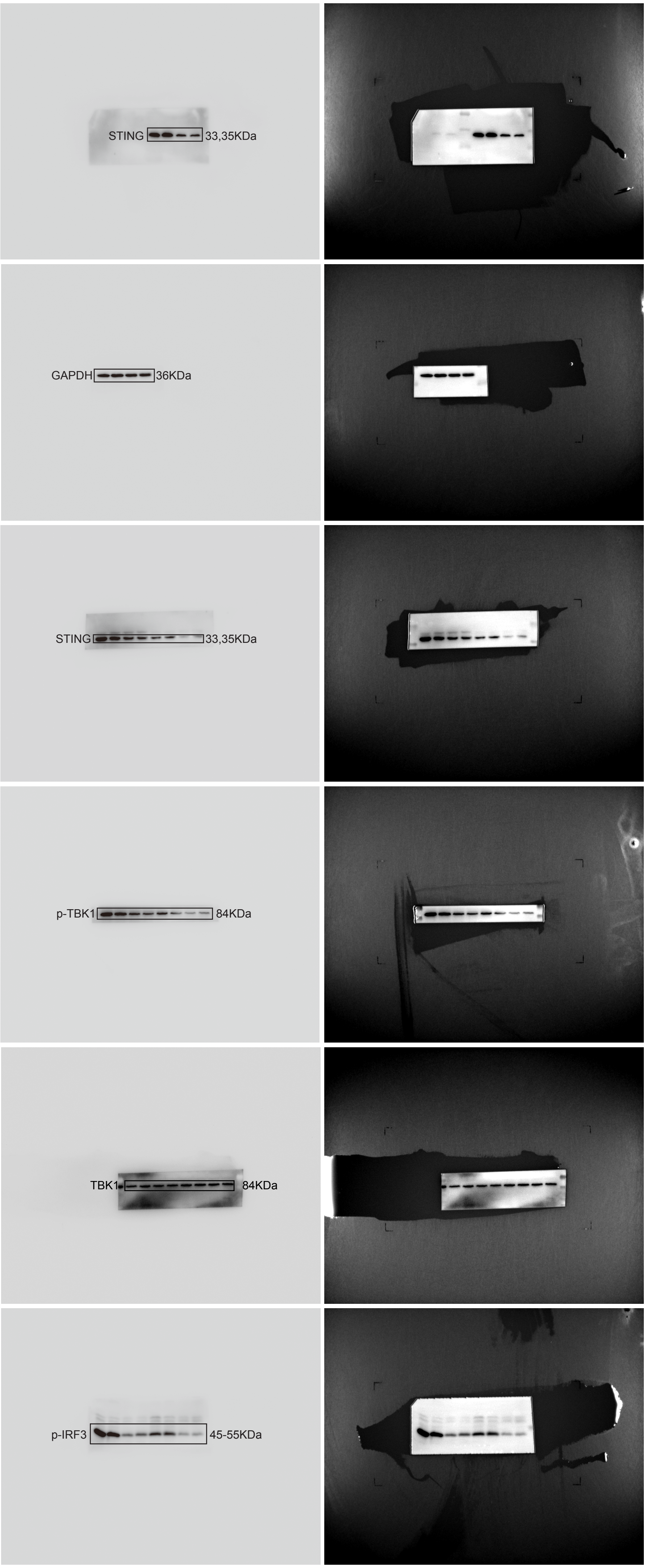

STING
33,35KDa
GAPDH
36KDa
STING
33,35KDa
p-TBK1
84KDa
TBK1
84KDa
p-IRF3
45-55KDa

Fig3.b

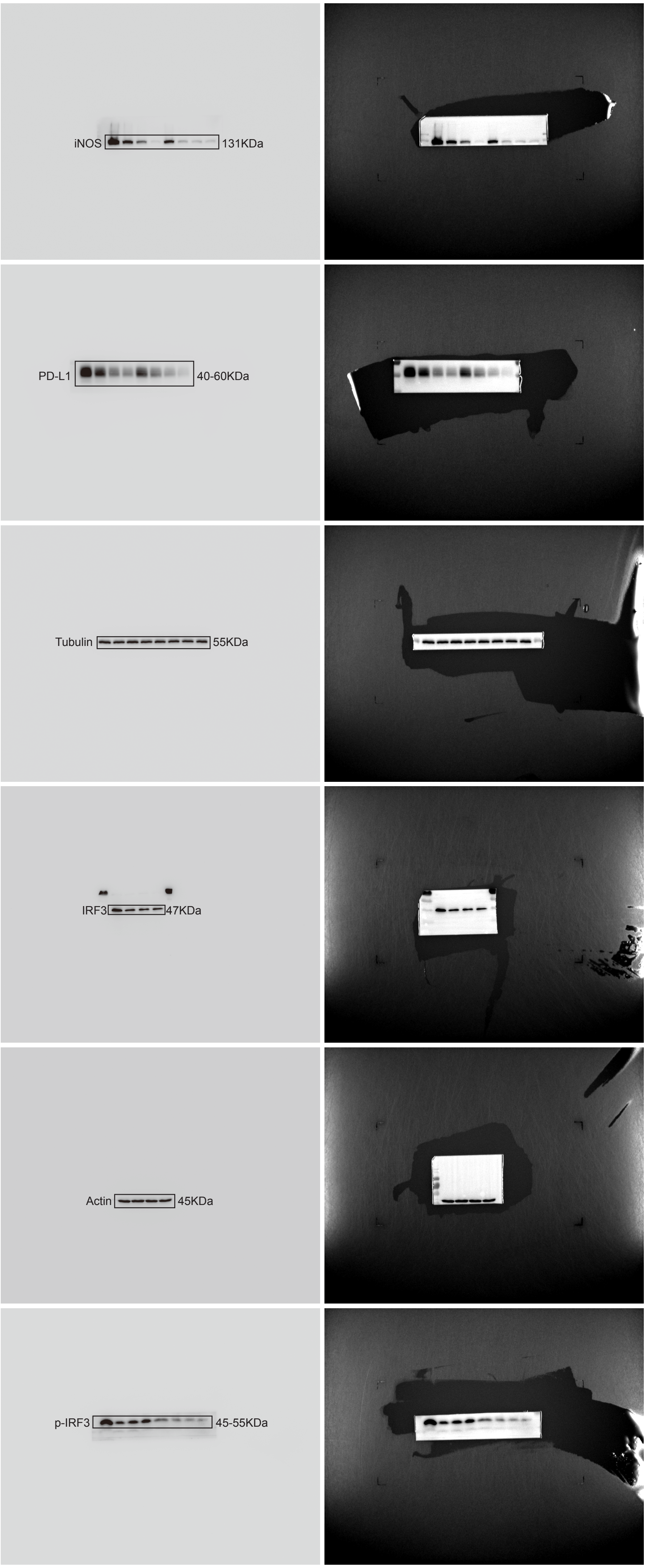
iNOS
131KDa
PD-L1
40-60KDa
Tubulin
55KDa
IRF3
47KDa
Actin
45KDa
p-IRF3
45-55KDa

Fig3.c

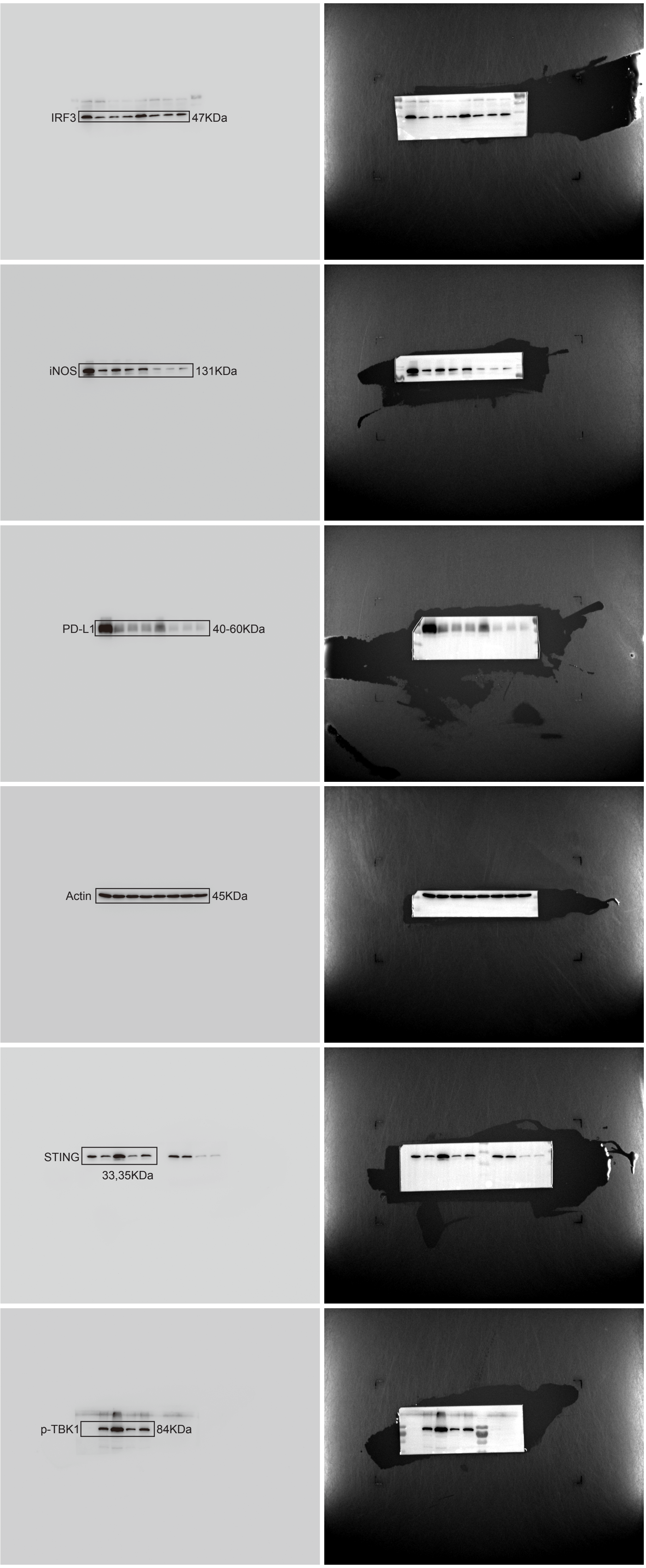
IRF3
47KDa
iNOS
131KDa
PD-L1
40-60KDa
Actin
45KDa
STING
33,35KDa
p-TBK1
84KDa

Fig3.d

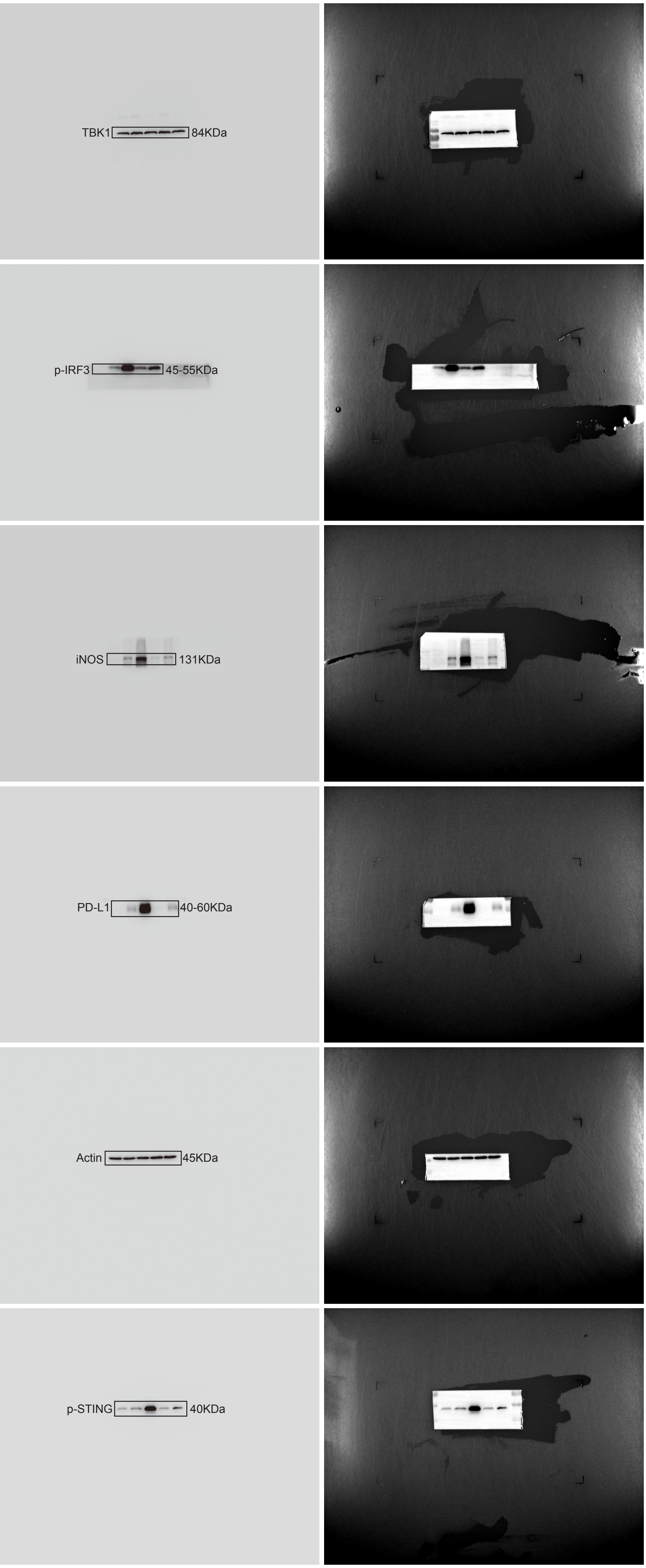

TBK1
84KDa
p-IRF3
45-55KDa
iNOS
131KDa
PD-L1
40-60KDa
Actin
45KDa
p-STING
40KDa

Fig3.e

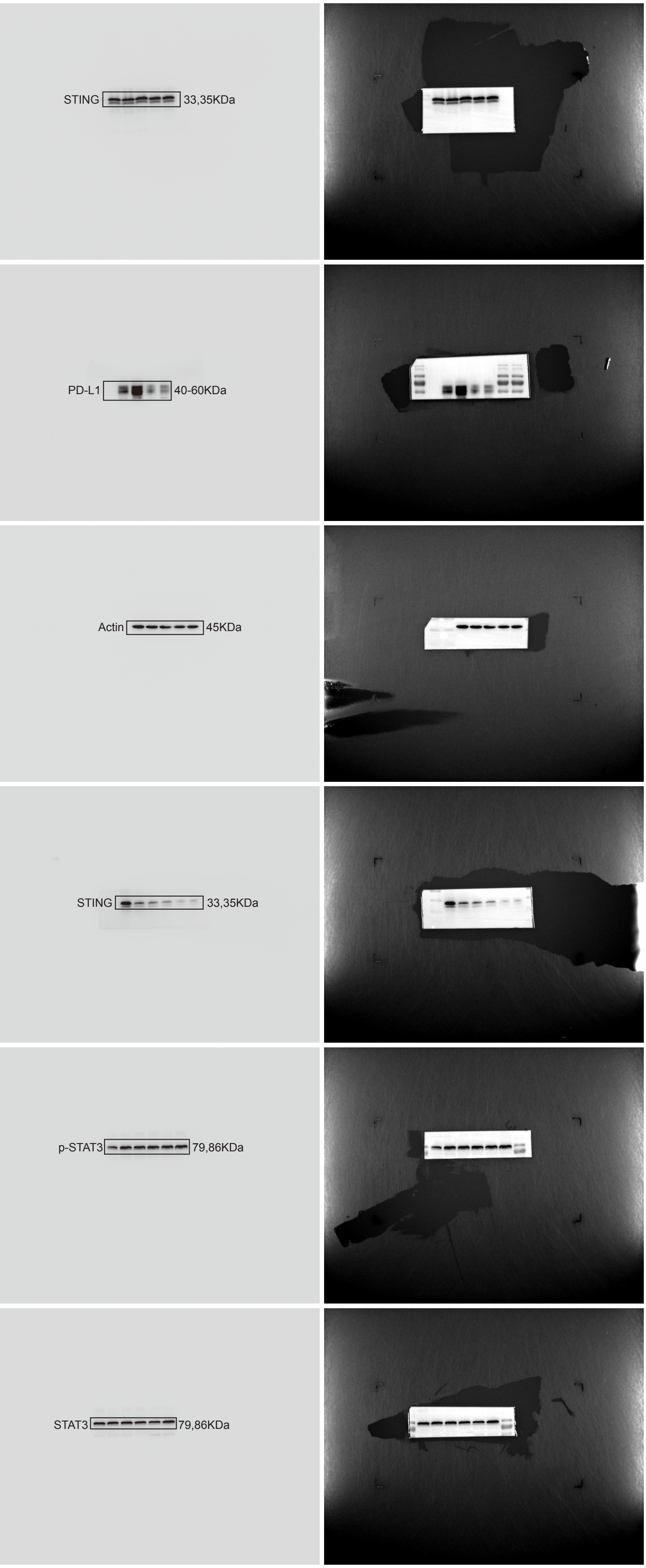
STING
33,35KDa
PD-L1
40-60KDa
Actin
45KDa
STING
33,35KDa
p-STAT3
79,86KDa
STAT3
79,86KDa

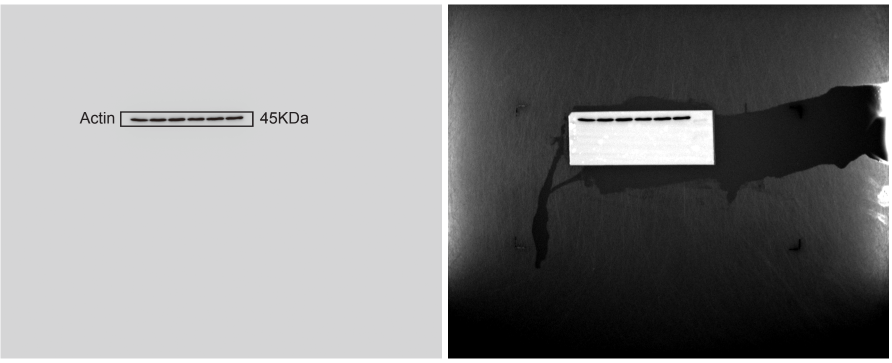
Actin
45KDa

Fig3.f

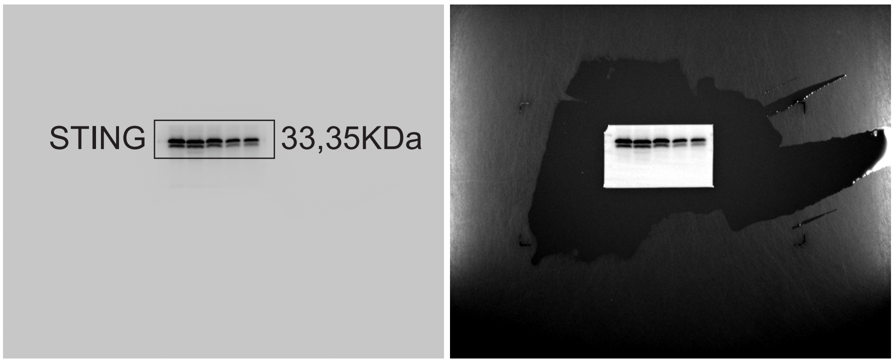
STING
33,35KDa

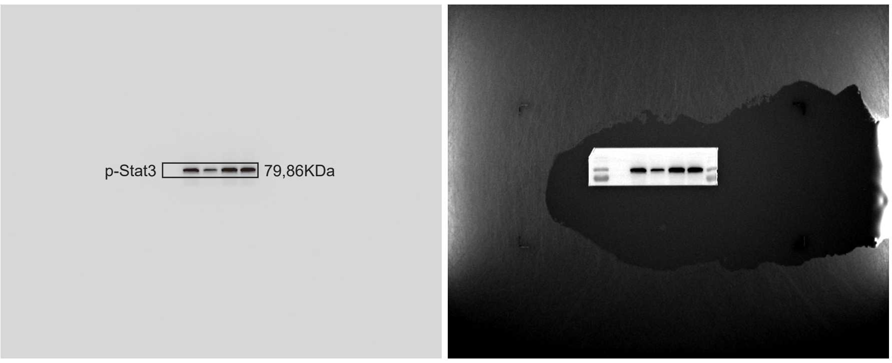
p-Stat3
79,86KDa

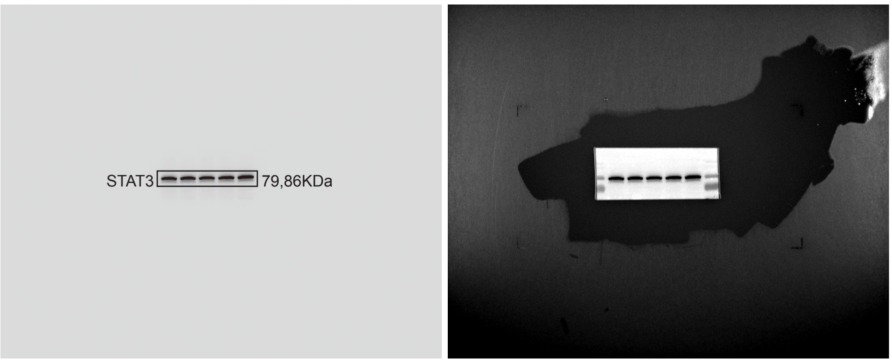
STAT3
79,86KDa

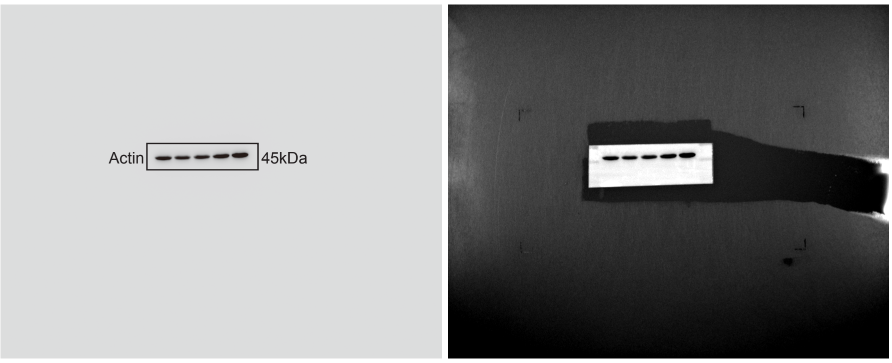
Actin
45kDa

Fig3.g

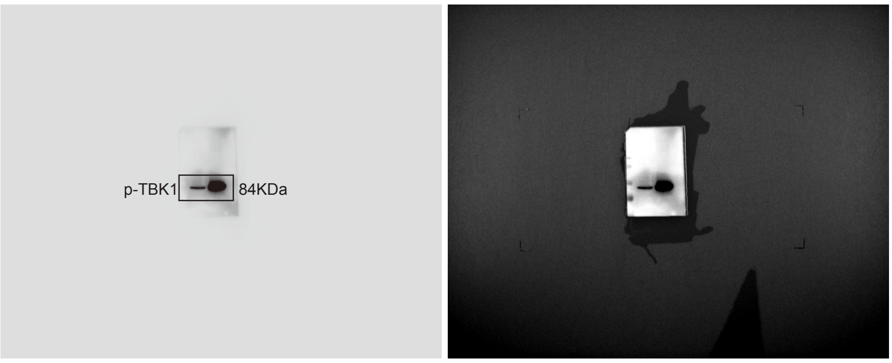
p-TBK1
84KDa

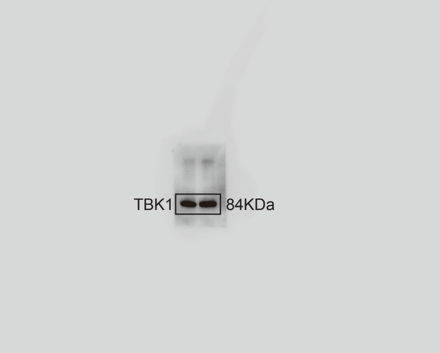
TBK1
84KDa
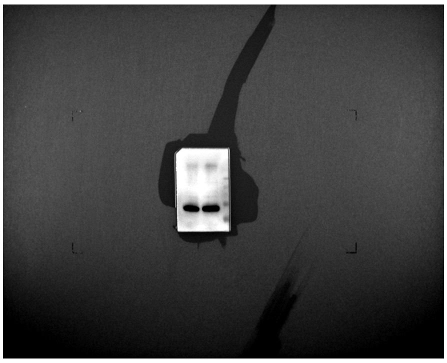
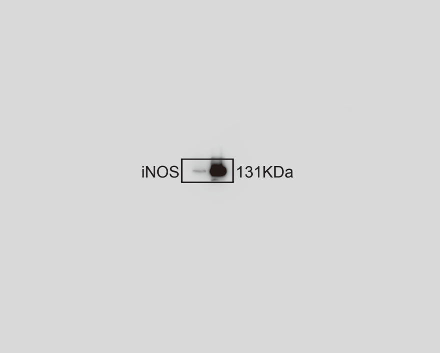
iNOS
131KDa
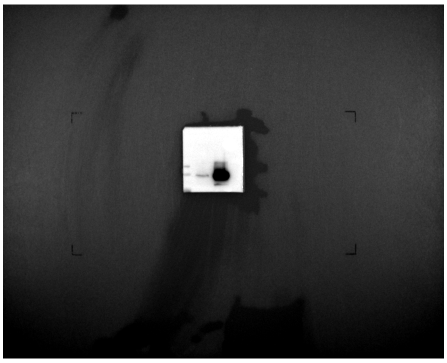
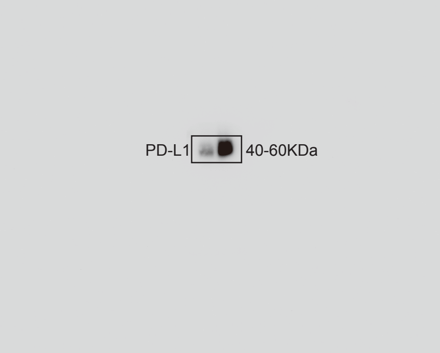
PD-L1
40-60KDa
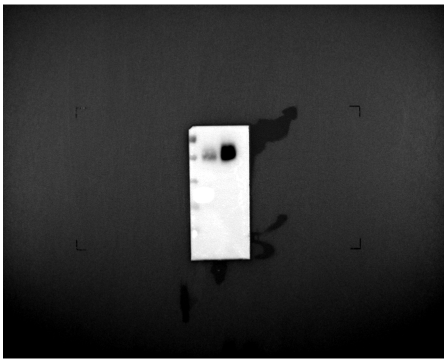
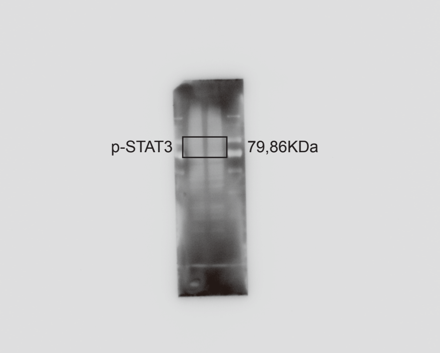
p-STAT3
79,86KDa
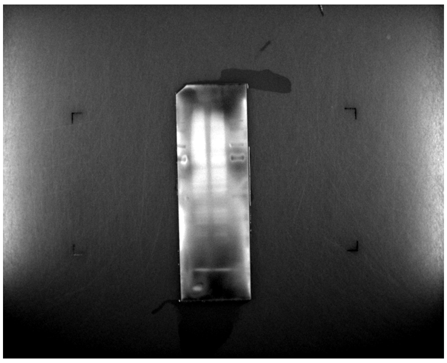
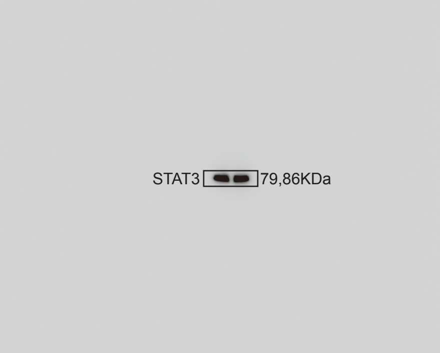
STAT3
79,86KDa
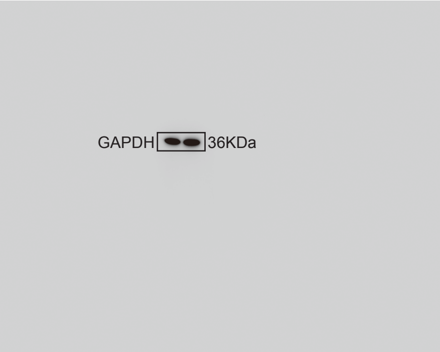
GAPDH
36KDa
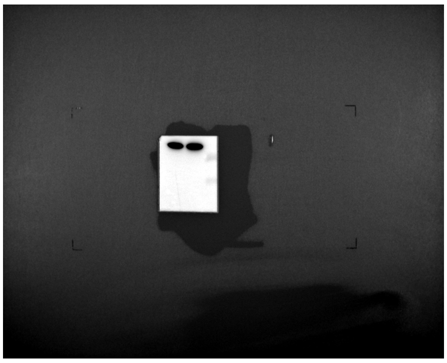

Fig3.h

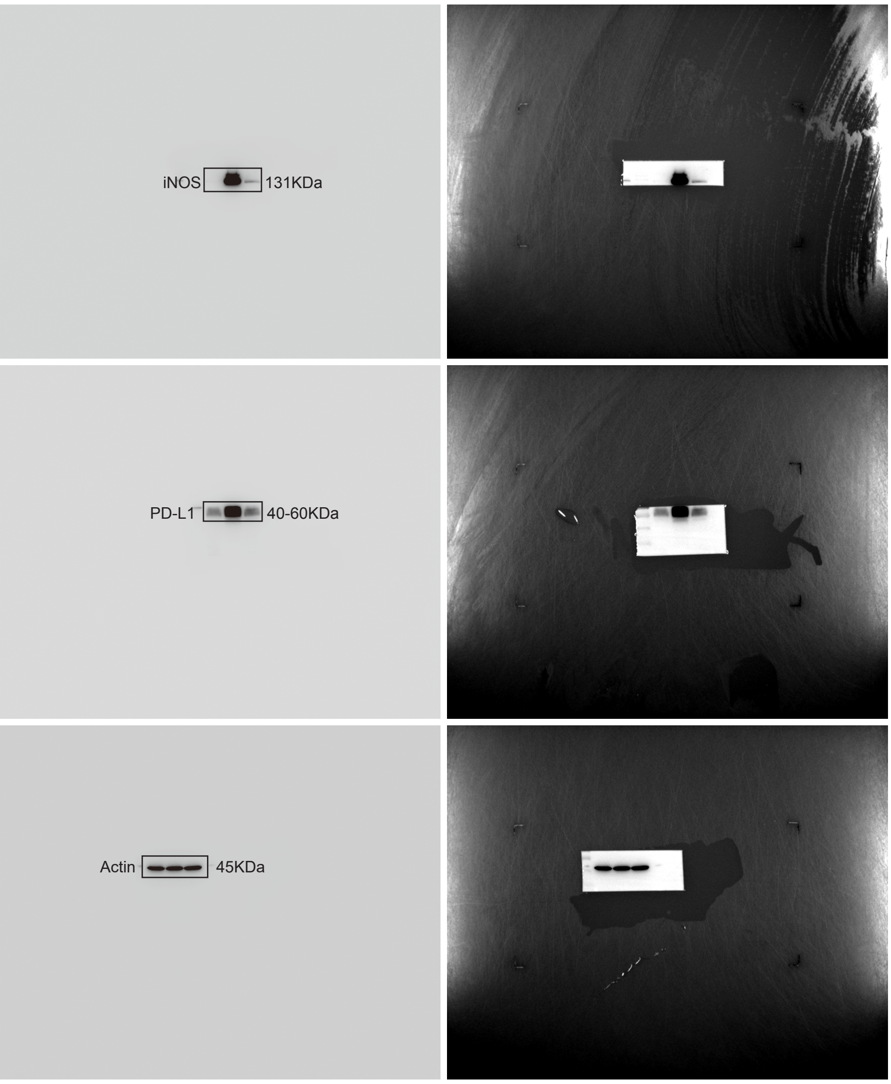
iNOS
131KDa
PD-L1
40-60KDa
Actin
45KDa

Fig3.i

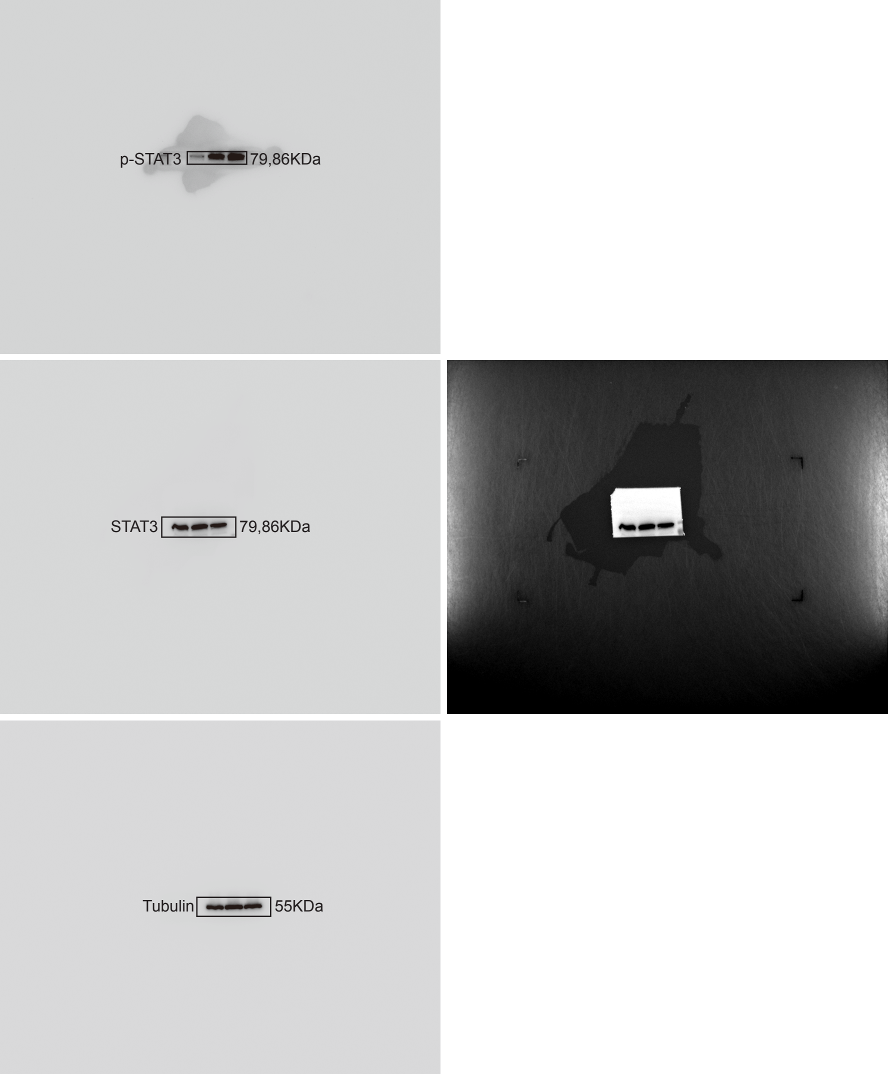
p-STAT3
79,86KDa
STAT3
79,86KDa
Tubulin
55KDa

Fig7.c

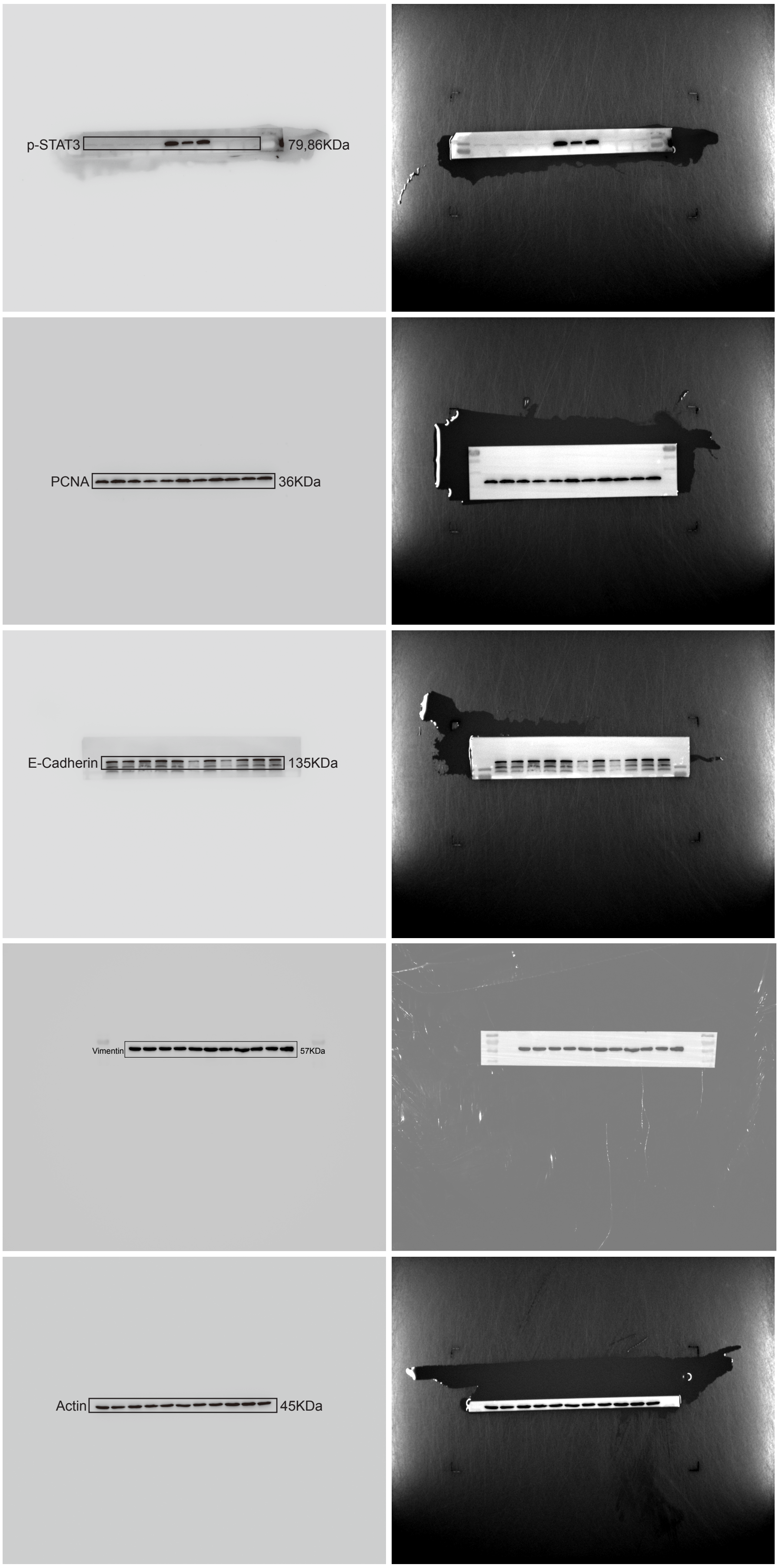
p-STAT3
79,86KDa
PCNA
36KDa
E-Cadherin
135KDa
Vimentin
57KDa
Actin
45KDa

Fig7.d

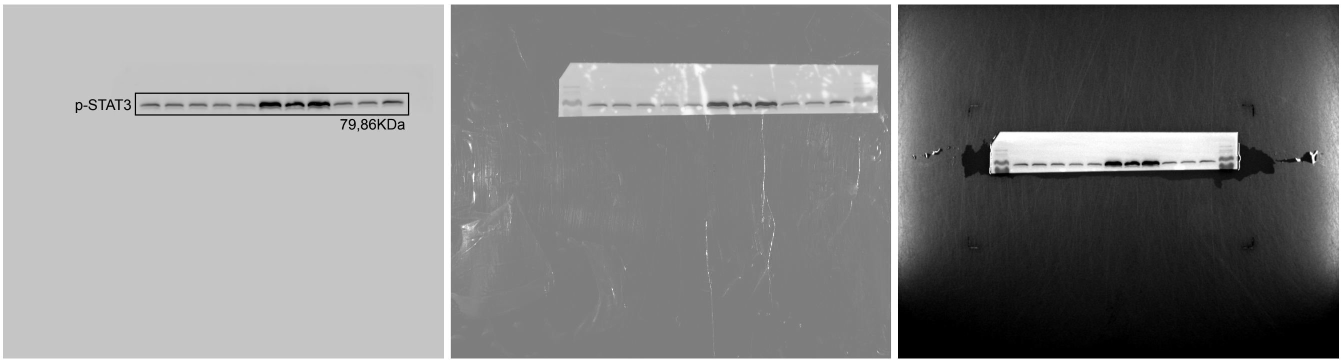
p-STAT3
79,86KDa

Fig7.e

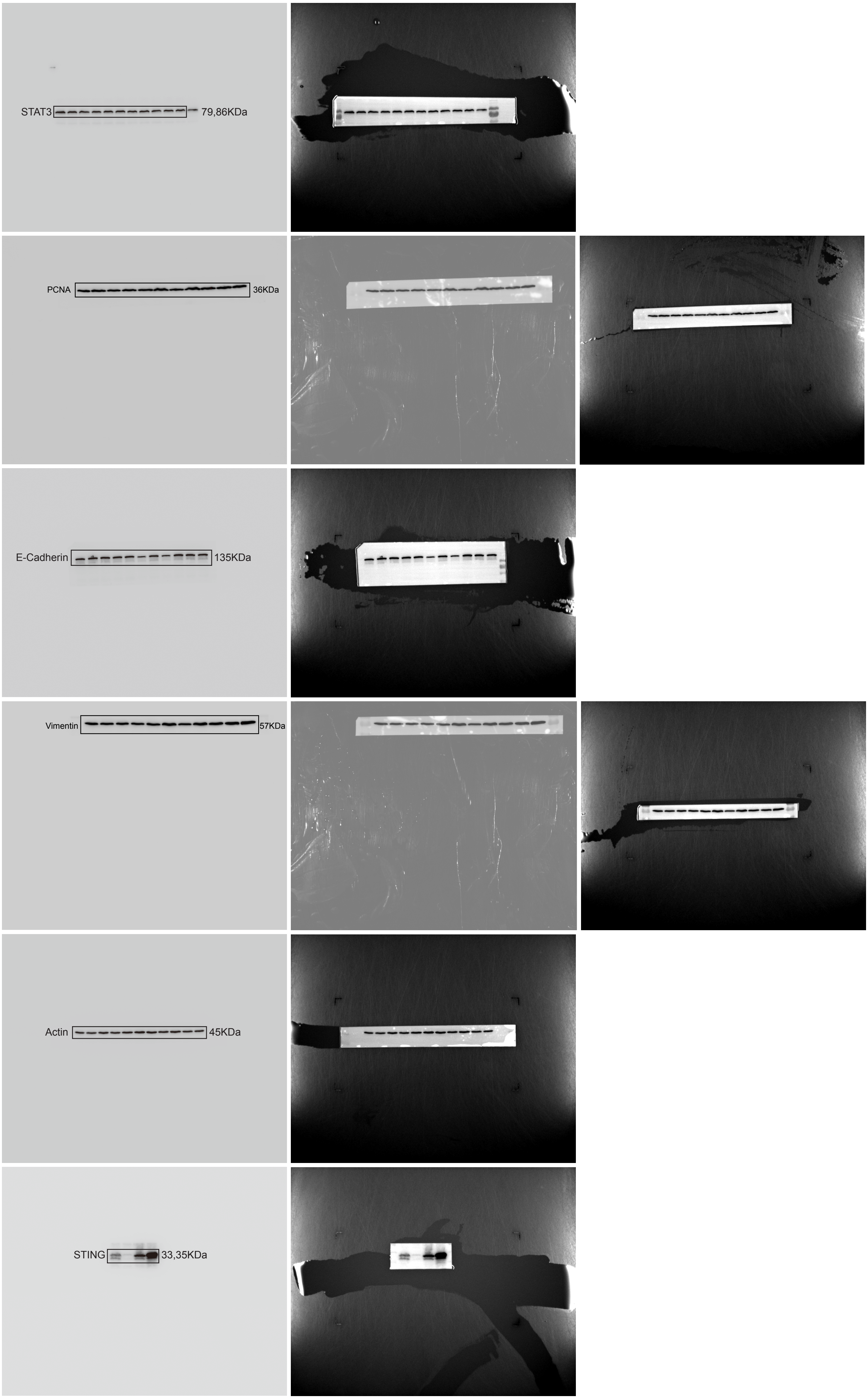
STAT3
79,86KDa
PCNA
36KDa
E-Cadherin
135KDa
Vimentin
57KDa
Actin
45KDa
STING
33,35KDa

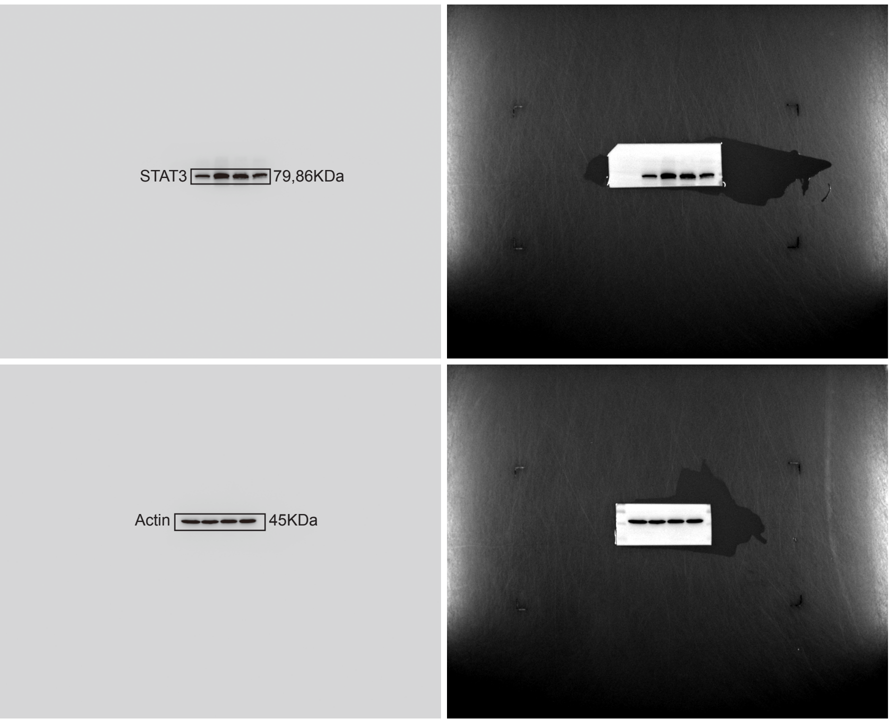
STAT3
79,86KDa
Actin
45KDa
